# Supplementary material for: Evaluation of freely available data profiling tools for health data research application: a functional evaluation review
Source: BMJ Open. 2022 May 9;12(5):e054186. doi: 10.1136/bmjopen-2021-054186 (PMC9086620; doi:10.1136/bmjopen-2021-054186)
Supplement: Supplementary data [file bmjopen-2021-054186supp001.pdf]

Supplemental Material 1. List of specific tools evaluated

Tool	Connectivity	Data Sources / File Formats
**Knime** (Data analytics, profiling, reporting and integration platform)	Connectivity to > 5 data sources	Simple text formats (CSV, PDF, XLS, JSON, XML, etc.)
		Unstructured data types (images, documents, networks, molecules, etc.)
		Time series data
		Connect to a host of databases and data warehouses to integrate data from Oracle, Microsoft SQL, Apache Hive, and more
		Load Avro, Parquet, or ORC files from HDFS, S3, or Azure
		Access and retrieve data from sources such as Twitter, AWS S3, Google Sheets, and Azure and extended via pandas
**Pandas Profiling (using Pandas I/O)** (Python module for exploratory data analysis (EDA))	Connectivity to > 5 data sources	Text: - CSV, fixed-width test files, JSON, HTML, Clipboard, Excel
		Binary: OpenDocument, HDF5 Format, Feather Format, Parqeuet Format, ORC Format, Msgpak, Stata, SAS, SPSS, Python Pickle Format
		SQL, Google BigQuery
**Orange** (Data visualization, machine learning, data profiling and mining toolkit)	Connectivity to > 5 data sources	Excel (.xlsx), simple tab-delimited (.txt), comma-separated files (.csv) or Google Sheets document
		distance matrix: Distance File
		predictive model: Load Model
		network: Network File from Network add-on
		images: Import Images from Image Analytics add-on
		several spectroscopy files: Multifile from Spectroscopy add-on
		PostgreSQL, SQL, online repository, and extended via pandas
**RapidMiner (LIMITED FREE VERSION)** (Integrated environment for data preparation, machine learning, deep learning, text mining, and predictive analytics)	Connectivity to > 5 data sources	Files: CSV, Stata, Hyper (Tableau), XLS, XML, QLikView, and more
		SQL: AccessDB, HSQLDB, Microsoft SQL Server (JTDS / Microsoft), MySQL, Oracle, PostgreSQL, Sybase
		NoSQL: Cassandra, MongoDB, Solr, Splunk (read only)
		Cloud services: Amazon S3, Azure blog and data lake, Dropbox, Google, Salesforce, Twitter, Zapier, Salesforce
**WEKA** (Machine learning	Connectivity to < 3 data sources	Arff, JSON, CSV, xrff, dat, data, names, and more
		Database using ODBC

**software to solve data mining problems)**		
**Anonimatron**<br><br>**(Pseudonymizes datasets)**	Connectivity to > 5 data sources	Oracle, PostgreSQL, MySQL, DB2, MsSQL, Cloudscape, Pointbase, Firebird, IDS, Informix, Enhydra, Interbase, Hypersonic, jTurbo, SQLServer and Sybase
**ARX Data Anonymization**<br><br>**(Scalable Data Anonymization Tool - supports multiple privacy models)**	Connectivity to > 5 data sources	CSV files, MS Excel spreadsheets
		Relational database systems, such as MS SQL, DB2, MySQL or PostgreSQL
**WhiteRabbit**<br><br>**(Tool to help prepare for ETLs of healthcare datasets)**	Connectivity to > 5 data sources	comma-separated text files
		MySQL, SQL Server, Oracle, PostgreSQL, Microsoft APS, Microsoft Access, Amazon RedShift, Google BigQuery
**Aggregate Profiler (AP)**<br><br>**(Data profiling and analysis tool)**	Connectivity to > 5 data sources	XML, XLS or CSV format, PDF export
		Teiid, Mysql, Oracle, Postgres, Access, Db2, SQL Server certified Big data support - HIVE
**Talend Open Studio for Data Integration (LIMITED FREE VERSION)**<br><br>**(Data integration and ETL)**	Connectivity to > 5 data sources	More than 900 pre-built connectors and components for Oracle, Teradata, Microsoft SQL server, Marketo, Salesforce, NetSuite, SAP, Microsoft Dynamics, Sugar CRM, Dropbox, Box, SMTP, FTP/SFTP, LDAP, and more
**Talend Open Studio for Big Data (LIMITED FREE VERSION)**<br><br>**(ETL for large and diverse data sets)**	Connectivity to > 5 data sources	Cloud: Amazon Web Services (AWS), Microsoft Azure, Google Cloud Platform, and more
		RDBMS: Oracle, Teradata, Microsoft SQL server, and more
		SaaS: Marketo, Salesforce, NetSuite, and more
		Packaged Apps: SAP, Microsoft Dynamics, Sugar CRM, and more
		Technologies: Dropbox, Box, SMTP, FTP/SFTP, LDAP, and more

**Talend Open Studio for Data Quality (LIMITED FREE VERSION)**<br><br>**(Assesses accuracy and integrity of data - Data Profiling Tool)**	Connectivity to > 5 data sources	Local or remote file that can be imported into the Talend Data Preparation tool (or from a database connection or other data sources, although not in the context of the Free Desktop version).<br>Excel or CSV file<br>90+ data sources and scale with Stitch Data Loader - https://www.talend.com/products/pricing-model/
**Talend Open Studio for ESB (LIMITED FREE VERSION)**	Connectivity to > 5 data sources	Cloud: Amazon Web Services (AWS), Microsoft Azure, Google Cloud Platform, and more<br>RDBMS: Oracle, Teradata, Microsoft SQL server, and more<br>SaaS: Marketo, Salesforce, NetSuite, and more<br>Packaged Apps: SAP, Microsoft Dynamics, Sugar CRM, and more<br>Technologies: Dropbox, Box, SMTP, FTP/SFTP, LDAP, and more
**Talend Open Studio for MDM (LIMITED FREE VERSION)**<br><br>**(key capabilities for data governance and master data management)**	Connectivity to > 5 data sources	AWS, Microsoft Azure, Google Cloud Platform, and more. Plus, SaaS, packaged apps, and web services
**OpenRefine**<br><br>**(Tool for cleaning and transforming data)**	Connectivity to < 3 data sources	TSV, CSV, *SV, .xls, .xlsx, JSON, XML, RDF as XML and google documents
**DataCleaner (COMMUNITY EDITION - Limited)**<br><br>**(Data profiling, data cleaning, and data integration tool) - offers integration with Pentaho**	Connectivity to > 5 data sources	CSV files, Excel spreadsheets<br>JDBC, MySQL, PostrgreSQL, SQL Server<br>Salesforce, SugarCRM
**DataPreparator**	Connectivity to < 3 data sources	JDBC, XLS

**(Preprocessing - data cleaning, transformation, and exploration)**		ARFF, DATA, CSV or plain text file format
**Data Match (30-DAY FREE TRIAL)**<br><br>**(visual data cleansing application - a component of Data Ladder)**	Connectivity to > 5 data sources	Access, Apache HBase, Dynamics CRM, Email, Excel, Facebook, JSON, MongoDB, MySQL, Salesforce, SugarCRM, Twitter, XML
**DataMartist (30 DAY FREE TRIAL, STANDARD - $349, PROFESSIONAL - $995)**<br><br>**(Visual, data profiling and data transformation tool)**	Connectivity to > 5 data sources	SQL Server, Oracle, MySQL, ODBC, MS Access, Excel Spreadsheets, Delimited text files including CSV data
**Pentaho Kettle (COMMUNITY EDITION - Limited)**<br><br>**(ETL Tool) Integrates with WEKA (Data Profiling)**	Connectivity to > 5 data sources	Oracle, PostgreSQL, Redshift, SAP, SQLite, SparkSQL, Sybase, Teradata, UniVerse, Verica, Cloudera Impala, Hypersonic, H2 and more
**SQL Power Architect (COMMUNITY EDITION - Limited)**<br><br>**(Data Modeling & Profiling Tool)**	Connectivity to > 5 data sources	JDBC, PostgreSQL, SQL, MySQL, HSSQLDB, Oracle, DB2, HSQLDB, SQLstream, H2, Derby
**SQL Power DqGuru**	Connectivity to > 5 data sources	JDBC, Oracle, Postgress, MySQL, Sybase and more

**(COMMUNITY EDITION - Limited)**<br><br>**(Data Cleansing & MDM Tool)**		
**DQ Analyzer (COMMUNITY EDITION - Limited)**<br>**(Data profiling tool)**	Connectivity to > 5 data sources	Oracle, MS SQL, DB2, Sybase, Teradata, MySQL, Apache Derby, PostgreSQL<br>CSV, TXT, and XLS(X)
**Pimcore**<br><br>**(Data Management, Integration, PIM, MDM, DAM)**	*Unable to collect during study*	*Unable to collect during study*
**CytoScape**<br><br>**(software platform for visualizing molecular interaction networks and biological pathways)**	*Unable to collect during study*	Simple interaction file (SIF or .sif format), Graph Markup Language (GML or .gml format), XGMML (extensible graph markup and modelling language), SBML, BioPAX, PSI-MI Level 1 and 2.5, Delimited text, Excel Workbook (.xls)
**Anaconda**<br><br>**(data science platform)**	Connectivity to > 5 data sources	Multiple Python Connectors
**Pyxplorer**<br><br>**(a simple tool that allows interactive profiling of datasets)**	Connectivity to < 5 data sources	Hive, Impala, MySQL
**MobyDQ**<br><br>**(Testing tool - aims to automate Data Quality checks**	Connectivity to > 5 data sources	Cloudera Hive, MariaDB, Microsoft SQL Server, MySQL, Oracle, PostgreSQL, SQLite, Teradata, Snowflake, Hortonworks Hive

**during data processing)**		
