# Supplementary material for: Evaluation of freely available data profiling tools for health data research application: a functional evaluation review
Source: BMJ Open. 2022 May 9;12(5):e054186. doi: 10.1136/bmjopen-2021-054186 (PMC9086620; doi:10.1136/bmjopen-2021-054186)
Supplement: Supplementary data [file bmjopen-2021-054186supp002.pdf]

Supplemental Material 2. A Data profiling report produced by Pandas Profiling (Python).

Overview

Overview | Reproduction | Warnings 32

Dataset statistics

**Number of variables**	21
**Number of observations**	10
**Missing cells**	5
**Missing cells (%)**	2.4%
**Duplicate rows**	0
**Duplicate rows (%)**	0.0%
**Total size in memory**	1.8 KiB
**Average record size in memory**	180.8 B

Variable types

**CAT**	14
**NUM**	7

CITY
Categorical

HIGH CORRELATION
MISSING

**Distinct count**	7
**Unique (%)**	77.8%
**Missing**	1
**Missing (%)**	10.0%
**Memory size**	80.0 B

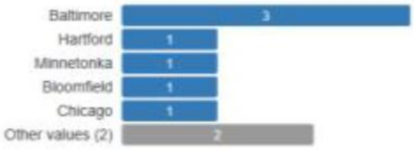

Toggle details

Common Values | Length

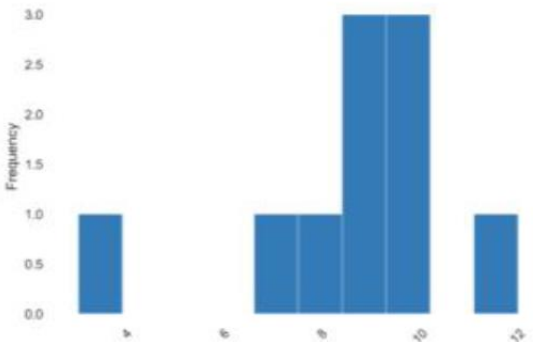

Length

**Max length**	12
**Median length**	9
**Mean length**	8.7
**Min length**	3

REVENUE

Real number ($\mathbb{R}_{\geq 0}$)

UNIQUE
ZEROS

Distinct count	10
Unique (%)	100.0%
Missing	0
Missing (%)	0.0%
Infinite	0
Infinite (%)	0.0%

Mean	87811405.0
Minimum	0.0
Maximum	154184100.0
Zeros	1
Zeros (%)	10.0%
Memory size	80.0 B

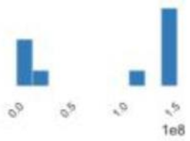

Toggle details

Statistics | Histogram(s) | Common values | Extreme values

Quantile statistics

Minimum	0
5-th percentile	587250
Q1	10433062.5
median	129576100
Q3	142068150
95-th percentile	153313215
Maximum	154184100
Range	154184100
Interquartile range (IQR)	131635087.5

Descriptive statistics

Standard deviation	70229707.73
Coefficient of variation (CV)	0.7997788867
Kurtosis	-2.177497116
Mean	87811405
Median Absolute Deviation (MAD)	23640350
Skewness	-0.4427638806
Sum	878114050
Variance	4.932211848e+15

Correlations

Pearson's r | Spearman's ρ | Kendall's τ | Phik (φk) | Cramér's V (φc)

Toggle correlation descriptions

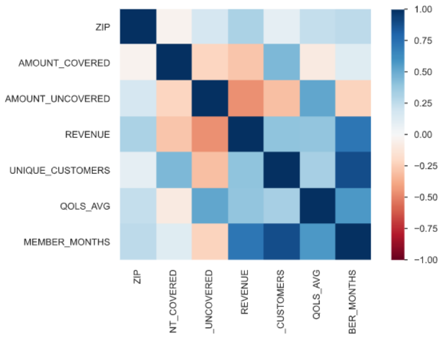

Pearson's r

The Pearson's correlation coefficient (*r*) is a measure of linear correlation between two variables. It's value lies between -1 and +1, -1 indicating total negative linear correlation, 0 indicating no linear correlation and 1 indicating total positive linear correlation. Furthermore, *r* is invariant under separate changes in location and scale of the two variables, implying that for a linear function the angle to the x-axis does not affect *r*.

To calculate *r* for two variables *X* and *Y*, one divides the covariance of *X* and *Y* by the product of their standard deviations.
