# Supplementary material for: Evaluation of freely available data profiling tools for health data research application: a functional evaluation review
Source: BMJ Open. 2022 May 9;12(5):e054186. doi: 10.1136/bmjopen-2021-054186 (PMC9086620; doi:10.1136/bmjopen-2021-054186)
Supplement: Author's manuscript [file bmjopen-2021-054186.draft_revisions.pdf]

BMJ Open is committed to open peer review. As part of this commitment we make the peer review history of every article we publish publicly available.

When an article is published we post the peer reviewers' comments and the authors' responses online. We also post the versions of the paper that were used during peer review. These are the versions that the peer review comments apply to.

The versions of the paper that follow are the versions that were submitted during the peer review process. They are not the versions of record or the final published versions. They should not be cited or distributed as the published version of this manuscript.

BMJ Open is an open access journal and the full, final, typeset and author-corrected version of record of the manuscript is available on our site with no access controls, subscription charges or pay-per-view fees (http://bmjopen.bmj.com).

If you have any questions on BMJ Open's open peer review process please email info.bmjopen@bmj.com

BMJ Open

Evaluation of Freely Available Data Profiling Tools for Health Data Research Application

Journal:	*BMJ Open*
Manuscript ID	bmjopen-2021-054186
Article Type:	Original research
Date Submitted by the Author:	04-Jun-2021

Complete List of Authors:	Gordon, Ben; Health Data Research UK<br>Fennessy, Clara; Health Data Research UK<br>Varma, Susheel; Health Data Research UK<br>Barrett, Jake; Health Data Research UK<br>McCondochie, Enez; Inspirata Ltd<br>Heritage, Trevor; Inspirata Ltd<br>Duroe, Oenone; Inspirata Ltd<br>Jeffery, Richard; Inspirata Ltd<br>Rajamani, Vishnu; Inspirata Ltd<br>Earlam, Kieran; Cystic Fibrosis Trust<br>Banda, Victor; Imperial College London Neonatal Medicine Research Group, Neonatal Data Analysis Unit<br>Sebire, Neil; Health Data Research UK

Keywords:	Information management < BIOTECHNOLOGY & BIOINFORMATICS, Health informatics < BIOTECHNOLOGY & BIOINFORMATICS, Information technology < BIOTECHNOLOGY & BIOINFORMATICS

SCHOLARONE™
Manuscripts

I, the Submitting Author has the right to grant and does grant on behalf of all authors of the Work (as defined in the below author licence), an exclusive licence and/or a non-exclusive licence for contributions from authors who are: i) UK Crown employees; ii) where BMJ has agreed a CC-BY licence shall apply, and/or iii) in accordance with the terms applicable for US Federal Government officers or employees acting as part of their official duties; on a worldwide, perpetual, irrevocable, royalty-free basis to BMJ Publishing Group Ltd ("BMJ") its licensees and where the relevant Journal is co-owned by BMJ to the co-owners of the Journal, to publish the Work in this journal and any other BMJ products and to exploit all rights, as set out in our licence.

The Submitting Author accepts and understands that any supply made under these terms is made by BMJ to the Submitting Author unless you are acting as an employee on behalf of your employer or a postgraduate student of an affiliated institution which is paying any applicable article publishing charge ("APC") for Open Access articles. Where the Submitting Author wishes to make the Work available on an Open Access basis (and intends to pay the relevant APC), the terms of reuse of such Open Access shall be governed by a Creative Commons licence – details of these licences and which Creative Commons licence will apply to this Work are set out in our licence referred to above.

Other than as permitted in any relevant BMJ Author's Self Archiving Policies, I confirm this Work has not been accepted for publication elsewhere, is not being considered for publication elsewhere and does not duplicate material already published. I confirm all authors consent to publication of this Work and authorise the granting of this licence.

For peer review only

Evaluation of Freely Available Data Profiling Tools for Health Data Research Application

Ben Gordon[1], Clara Fennessy[1], Susheel Varma[1], Jake Barrett[1], Enez McCondochie[2], Trevor Heritage[2], Oenone Duroe[2], Richard Jeffery[2], Vishnu Rajamani[2], Kieran Earlam[3], Victor Banda[4], Neil J Sebire[1]

1. Health Data Research UK, London, UK
2. Inspirata Ltd, Tampa, Florida, USA
3. Cystic Fibrosis Trust, London, UK
4. Neonatal Data Analysis Unit, Imperial College London, London, UK

Correspondence:

Professor Neil J Sebire

Chief Clinical Data Officer, Health Data Research UK

Wellcome Trust, Gibbs Building, 215 Euston Road, London, NW1 2BE

Email: neil.sebire@hdruk.ac.uk

Word Count: 2887

For peer review only

For peer review only - http://bmjopen.bmj.com/site/about/guidelines.xhtml

ABSTRACT

Objectives: To objectively evaluate freely available data profiling software tools using healthcare data.

Design: Data profiling tools were evaluated for their capabilities using publicly available information and data sheets. From initial assessment, several underwent further detailed evaluation for application on healthcare data using a synthetic dataset of 1000 patients and associated data using a common health data model, and tools scored based on their functionality with this dataset.

Setting: Improving the quality of healthcare data for research use is a priority. Profiling tools can assist by evaluating datasets across a range of quality dimensions. Several freely available software packages with profiling capabilities are available but healthcare organizations often have limited data engineering capability and expertise.

Participants: 28 profiling tools, eight undergoing evaluation on synthetic dataset of 1000 patients.

Results: Of 28 potential profiling tools initially identified, eight showed high potential for applicability with healthcare datasets based on available documentation, of which two performed consistently well for these purposes across multiple tasks including determination of completeness, consistency, uniqueness, validity, accuracy and provision of distribution metrics.

Conclusions: Numerous freely available profiling tools are serviceable for potential use with health datasets, of which at least two demonstrated high performance across a range of technical data quality dimensions based on testing with synthetic health dataset and common data model. The appropriate tool choice depends on factors including underlying organizational infrastructure, level of data engineering and coding expertise, but there are

For peer review only

For peer review only - http://bmjopen.bmj.com/site/about/guidelines.xhtml

freely available tools helping profile health datasets for research use and inform curation activity.

For peer review only

For peer review only - http://bmjopen.bmj.com/site/about/guidelines.xhtml

Strengths and limitations of this study

- We are not aware of any other publication reviewing open and open-source data profiling tools using this level of rigour.
- A range of freely available data profiling tools are capability mapped regarding utility for profiling health data sets.
- Use of such data profiling software tools can help improve data quality by understanding the technical dimensions of a given health data set
- There may be other potentially suitable tools in existence that were not discovered and evaluated.
- It was not always possible to find out information on individual tools from available documentation.

For peer review only

For peer review only - http://bmjopen.bmj.com/site/about/guidelines.xhtml

INTRODUCTION

HDR UK's mission is to unite the UK's health data to enable discoveries that improve people's lives.[1] One aspect of this activity is the ambition to provide a consistent view on the utility of particular datasets for specific purposes through an Innovation Gateway.[2] This would allow users to understand whether a dataset is likely to meet their needs, ahead of requesting access. One important aspect of the utility of a dataset relates to the technical dimensions of data quality,[3] as the consistent use of data quality metrics can facilitate comparison between datasets and, in addition, can demonstrate areas of potential improvement for data custodians. Commonly used data quality dimensions include completeness, consistency, uniqueness, validity, accuracy, and timeliness.

In addition to domain-specific subject matter expertise, semi-automated analysis of datasets using data quality profiling software tools can assist the process, supporting increased awareness of data quality of datasets, completeness and consistency of data submissions, improved reliability, accuracy and auditability and ultimately 'better' more usable data over time. Data profiling is the process of reviewing source data, understanding the structure, content and interrelationships of elements, examining records to discover errors/issues relating to content and format, and understanding data distributions and other factors.[4] It is seen as an important step towards improving the quality and usefulness of data.[5] There are many challenges in profiling data, depending on the structure and format of the underlying data.[6]

Many software tools are available, with varied applicability and data profiling capability for healthcare data. The aims of this study were to identify and evaluate functionality and usability of existing openly available (either open source or free-to-use) data quality assessment tools for potential users across the health data research community with specific focus on data profiling capabilities.

For peer review only - http://bmjopen.bmj.com/site/about/guidelines.xhtml

METHODS

Study design

In order to evaluate existing freely available data profiling tools for potential use with health datasets, a desk-based activity was performed. This first required the identification of as many tools as possible that would be available without cost, followed by an initial evaluation of the identified tools against a range of broad criteria based on publicly available information regarding the tool functionalities. Following this evaluation, tools which scored highly in the areas of most interest for profiling of health datasets were tested on a synthetic health dataset to evaluate their capability in an objective way.

Identification of tools

An initial scoping exercise was conducted to identify data profiling tools that were freely available. This included tools that were open-source and those that were proprietary but freely available (or having a functional freely available version). This involved web searches, supplemented by discussion with individuals currently working in the sector and involved in data profiling and curation. The inclusion criteria were based on license restrictions, cost, lack of expert level user requirements and appropriateness of functionality as relates to health data quality, resulting in 28 potential tools for initial evaluation.

Initial Evaluation

In order to evaluate the tools, a general comparison matrix was developed based on criteria used previously for evaluating data quality tools.[7] The 28 tools were initially compared and categorized against the matrix using information from the available product documentation and data sheets. The scoring matrix was developed as a feature tree, comprising five major functional areas and fourteen minor functional areas, and a maximum score allocated for each area.(Table 1)

For peer review only - http://bmjopen.bmj.com/site/about/guidelines.xhtml

Table 1. Detailed Scoring Criteria per Feature

FEATURE TREE					SCORE
**Data Ingestion and Integration**	→	*Data Consolidation*	→	Connectivity to N data sources	5
			→	(ETL) Data Extraction, Transformation and Loading / ETL and ELT support	5
			→	Data Modelling	5
	→	*Data Propagation*	→	Data flow orchestration, Enterprise application integration (EAI), exchange of messages and transactions	5
			→	Enterprise data replication (EDR), transfer large amounts of data between databases	5
			→	Versioning and file management	5
	→	*Data Virtualization*	→	Data Access	5
	→	*Data Federation*	→	Enterprise information integration (EII)	5
				**Total**	**40**
**Data Preparation and Cleaning**	→	*Parsing and Standardization*	→	Tagging data with keywords, descriptions or categories	5
			→	Data Scrubbing/Cleansing/Handling blank values/Reformatting values/Threshold checking	5
			→	Data Enhancement/Enrichment/Curation	5
			→	NLP	5
			→	Address validation/geocoding	5
			→	Master Data Management	5
			→	Data masking	5
	→	*Identity Resolution, Linkage, Merging & Consolidation*	→	Data Deduping	5
			→	Machine Learning / Training a statistical model	5
			→	Data aggregation	5
			→	Data Binning	5
			→	Grouping similar data / Clustering	5
			→	Outlier detection and removal	5
	→	*Master Reference Data Management*	→	"Hub" infrastructure to source and distribute master/reference data	5
			→	Master data versioning based on data history and timelines	5
			→	Workflow integrations to steward and publish the master/reference data	5
			→	Graph data stores to define relationships for creating a flexible knowledge graph	5
			→	Accessible API for real-time access to shared reference data	5
				**Total**	**90**
**Data Profiling, Exploration/**	→	*Relationship discovery*	→	Cross Table Redundancy Analysis	5
			→	Performing data quality assessment, risk of performing joins on the data	5

For peer review only - http://bmjopen.bmj.com/site/about/guidelines.xhtml

Category	Sub-category	Feature	Score
**Pattern Detection**		Identifying distributions, key candidates, foreign-key candidates, functional dependencies, embedded value dependencies, and performing inter-table analysis.	5
	Content discovery	Data Pattern Discovery	5
		Domain Analysis	5
		Discovering metadata and assessing its accuracy	5
	Structure discovery	Column Value Frequency Analysis & Statistics, collecting descriptive statistics like min, max, count and sum.	5
		Table Structure Analysis, Collecting data types, length and recurring patterns.	5
		Drill-through Analysis	5
		**Total**	**45**
**Data Monitoring**	*Monitoring & Alerting*	Time series data identified and collection by metric name and key/value pairs	5
		Flexible query language to leverage this dimensionality	5
		Graphing and dashboarding support	5
		**Total**	**15**
**Data Use**	*Metadata Management*	Concept Identification and Naming	5
		Data Categorization	5
		Lineage	5
		Relationship with other metadata	5
		Comments and Remarks	5
		Data Stats (profiles)	5
		Knowledge Graph	5
	Privacy & Security	Data Anonymization	5
		Role based access control	5
		Secure environment setup and deployment	5
		Container based deployment	5
	Data Mining	Interactive Data Visualization	5
		Visual Programming and analysis	5
		Visual Illustrations & training documentation	5
		Sample Data / Generate Fake Data	5
		Add-ons and Extension Functionality	5
		**Total**	**80**

Each tool was ranked based on key capabilities required to address the profiling aspects of data quality using the feature tree and scoring. Tools were assigned the available weighted scoring based on the ability to provide the function described, according to the information available. Each feature was scored using a binary system, either 0 or 5. An exception to this

For peer review only

For peer review only - http://bmjopen.bmj.com/site/about/guidelines.xhtml

rule is the "Connectivity to N data sources" where this feature is scored 3, 4, and 5 when a tool has connectivity to < 3, < 6, and > 5 data sources, respectively. Scores for each of the five major category areas were converted to a percentage of the total available score for that area.

In-depth evaluation

Following the initial evaluation, eight tools scored were selected for further, in-depth evaluation based on the data profiling major category score and functions (the focus of this process was to evaluate data profiling capabilities; other potential functionalities were recorded for interest as above but not used for ranking). The selected tools included: Knime, DataCleaner, Orange, WEKA, Pandas-profiling (Python), Aggregate Profiler, Talend Open Studio for Data Quality, WhiteRabbit. (Rapid Miner and DQ Analyzer were excluded since they were limited free versions of paid-for tools. Since two python tools, Pandas Profiling and Anaconda, scored highly for profiling, only Pandas profiling was further evaluated since it is explicitly intended for data profiling. Finally, WhiteRabbit, Talend Open Studio for Data Quality and Aggregate Profiler were also evaluated since they were identified as being used by the HDR UK community). To evaluate these tools for their data profiling performance and capability, synthetic data sets were created using the open source tool, Synthea to generate CSV files and SQL Database adhering to the OMOP data model containing 1000 patients and related clinical data and the tools run on this dataset. Synthea allows generation of fully synthetic datasets which broadly conform to the data types and values expected in a 'real' health dataset but with no risk of patient data identification.[8] To evaluate performance and scalability of each tool an additional synthetic dataset of 1.3 million records was also generated.

Each of the specified open-source data profiling tools were evaluated based on how possible it was to execute common specific profiling functions as described in the tool documentation decided based on the Gartner reports.[9]

Further to this, the tools were evaluated based on the ability to deliver data profiles against core DAMA UK data quality dimensions,[3][10] including completeness (the proportion of stored data against the potential of 100% complete), consistency (the absence of difference,

For peer review only - http://bmjopen.bmj.com/site/about/guidelines.xhtml

when comparing two or more representations of a thing against a definition), uniqueness (nothing recorded more than once based upon how that thing is identified), validity (data are valid if it conforms to the syntax (format, type, range) of its definition), accuracy (the degree to which data correctly describes the object or event being described) and timeliness (the degree to which data represent reality from the required point in time). For each data profiling functionality, tools were run and subjectively scored on a scale of 0-5 according to a semi-structured scale (0=unable to process, 1=most requirements not achieved, 2=some requirements not achieved, 3=meets core requirements, 4=meets and exceeds some requirements, 5=significantly exceeds core requirements).

The suitability of the tools for potential future use by other parties was estimated based on feedback from volunteers from the HDR UK community testing selected tools on their local datasets and providing a qualitative comment on usability. Formal evaluation of the tools of a range of real-world health datasets in a range of environments was outside the scope of this study.

Patient and Public Involvement

Patients and/or the public were not involved in the design, or conduct, or reporting, or dissemination plans of this research.

For peer review only

For peer review only - http://bmjopen.bmj.com/site/about/guidelines.xhtml

RESULTS

Initial evaluation

The initial 28 tools evaluated are shown in Online Supplemental Material 1 along with scores in the various data quality task categories with detailed results for data profiling functionality. The overall results of the initial scoring are shown in Figure 1.

Subsequent evaluation

Based on the review of the tools to evaluate their ability to deliver key functions, the Python library, Pandas Profiling, was identified as possessing the most versatile functionality, able to complete all 30 of the identified profiling functions on the synthetic dataset for testing. The next most versatile tool, Knime, was able to perform 19 such tasks. Across the functionality types, Single Column – Cardinalities was one that the most tools were capable of delivering, with all tools able to deliver three of the functions in this type. The functionality type that was least well served by the tools was Dependencies, with only Pandas Profiling able to deliver any of these functions.(Table 2)

Table 2. Specific Data Profiling Tool Functionalities Evaluated

*** Key:**
K=Knime; DC=DataCleaner; O=Orange; W=WEKA; PP=Pandas Profiling (Python); AP=Aggregate Profiler; TOS=Talend Open Studio for Data Quality; WR=WhiteRabbit

FUNCTIONALITY TYPE	FUNCTION	DATA PROFILING TOOLS CAPABLE OF NATIVELY EXECUTING FUNCTION *							
		K	DC	O	W	PP	AP	TOS	WR
**Single Column – Cardinalities** REFERS TO THE UNIQUENESS OF DATA VALUES CONTAINED IN A PARTICULAR COLUMN (ATTRIBUTE) OF A TABLE (ENTITY)	*Number of rows*	✓	✓	✓	✓	✓	✓	✓	✓
	Number of nulls	✓	✓	✓	✓	✓	✓	✓	✓
	Percentage of nulls	✓		✓	✓	✓		✓	✓
	Number of distinct values (cardinality)	✓	✓	✓	✓	✓	✓	✓	✓
	Percentage of distinct values (Number of distinct values divided by the number of rows)	✓			✓	✓		✓	
**Single Column - Value distributions** PRESENTS AN ORDERING OF THE RELATIVE FREQUENCY (COUNT AND PERCENTAGE) OF THE ASSIGNMENT OF DISTINCT VALUES	*Frequency histograms (equi-width, equi-depth, etc.)*	✓				✓			
	Minimum and maximum values in a numeric column	✓	✓	✓		✓	✓	✓	✓
	Constancy (Frequency of most frequent value divided by number of rows)	✓				✓		✓	

For peer review only

For peer review only - http://bmjopen.bmj.com/site/about/guidelines.xhtml

	Quartiles (3 points that divide the numeric values into 4 equal groups)	✓	✓			✓	✓	✓	✓
	Distribution of first digit in numeric values (to check Benford's law)	✓				✓		✓	
**Single Column - Patterns, datatypes, and domains** REFERS TO THE DISCOVERY OF PATTERNS AND DATA TYPES	*Basic types (e.g., numeric, alphanumeric, date, time)*	✓				✓			
	DBMS-specific data type (e.g., varchar, timestamp)	✓	✓			✓	✓	✓	✓
	Measurement of Value length (minimum, maximum, average, median)	✓	✓	✓		✓	✓		✓
	Maximum number of digits in numeric values	✓	✓			✓	✓		
	Maximum number of decimals in numeric values	✓				✓	✓		
	Histogram of value patterns (Aa9...)	✓	✓			✓		✓	
	Generic semantic data type (e.g., code, date/time, quantity, identifier)	✓	✓			✓		✓	
	Semantic domain (e.g., credit card, first name, city)	✓	✓			✓		✓	
**Dependencies** DETERMINES THE DEPENDENT RELATIONSHIPS WITHIN A DATA SET	*Unique column combinations (UCCs) (key discovery)*					✓			
	Relaxed unique column combinations					✓			
	Inclusion dependencies (INDs) (foreign key discovery)					✓			
	Relaxed inclusion dependencies					✓			
	Functional dependencies					✓			
	Conditional functional dependencies					✓			
**Advanced Multi Column profiling** DETERMINES THE SIMILARITIES AND DIFFERENCES IN SYNTAX AND DATA TYPES BETWEEN TABLES (ENTITIES) TO DETERMINE WHICH DATA MIGHT BE REDUNDANT AND WHICH COULD BE MAPPED TOGETHER	*Correlation analysis*			✓		✓	✓		
	Association rule mining					✓			
	Cluster analysis					✓			
	Outlier detection	✓		✓		✓			
	Exact duplicate tuple detection		✓			✓		✓	
	Relaxed duplicate tuple detection		✓			✓		✓	
	**Total**	**19**	**13**	**8**	**5**	**30**	**10**	**15**	**8**

The tools were further evaluated based on their ability to deliver data profiles against the DAMA dimensions.(Figure 2) Pandas Profiling achieved significantly greater results compared to the other tools, scoring 110 of the available points, compared to the next highest tool, Knime, with 61 points. Of the tools examined, WhiteRabbit had the least comprehensive

For peer review only - http://bmjopen.bmj.com/site/about/guidelines.xhtml

functionality in this area, able only to provide information against the Completeness element. Across the different elements, Completeness was best served by the profiling tools, with all tools able to provide some functionality in this area. The least well-served element was Consistency, with only Pandas Profiling able to provide any output for this element. Online Supplemental Material 2 shows the profile reporting information produced by Pandas Profiling with features including basic dataset statistics overview, reports on specific numerical or categorical variables, and correlations between variables.

Links for all tools tested are available here (https://github.com/HDRUK/data-utility-tools).

User testing feedback

To provide anecdotal feedback on the usability of the tools, five of the eight tools (DataCleaner, Orange, MobyDQ, Knime and Aggregate profiler) were tested by volunteers from the Cystic Fibrosis Trust and the Neonatal Medicine Research Group. These tools were selected for testing based of the volunteer's ability and the resources available to run them.

MobyDQ and Aggregate Profiler both presented difficulties to the volunteers due to challenges installing and running the software. MobyDQ failed to authenticate due to issues with private keys and Aggregate Profiler crashed upon attempts to update.

Knime, DataCleaner and Orange could be run successfully by the volunteers. Orange required the local migration of data and installation of two additional modules, and was supported more effectively on Mac OS and Linux than Windows. Knime was fairly resource intensive and initially difficult to use, but was seen to be capable of a range of functions. DataCleaner was reported to be relatively easy to set up and run, even on a Windows machine, and capable of linking to existing databases.

For peer review only - http://bmjopen.bmj.com/site/about/guidelines.xhtml

DISCUSSION

The findings of the present study have demonstrated that numerous openly available data profiling tools are available, with several able to perform well using health datasets. The precise choice of tool for organisations will depend on the data type, model and format, in addition to IT environment, such as Windows or Linux, and expertise with such tools and coding languages, such as Python. Regardless of the tools used, appropriate deployment and dataset evaluation through data profiling should lead to early detection of data quality issues for particular data sets and sources and consequent ability to remediate such issues. The identification of Pandas Profiling as a versatile approach to data profiling is reinforced by the fact that, as a Python library, it can be combined with other tools, such as Orange or Knime, to provide an even more in-depth output.

This study provides a useful resource for individuals anywhere in the world to understand the functionality of freely available data profiling tools for use with health datasets, and put these to use. The creation of an open and persistent resource is a strength of the study. All the outputs of the testing, as well as the generated dataset, are available (https://github.com/HDRUK/data-utility-tools). None of the tested tools are specific to health data, and therefore could be used in any other domain. However, the open nature of the search for the tools, the absence of an indexed repository of these tools was likely non-exhaustive. There may be additional tools that would also have been suitable for this exercise that were not identified during the project. Furthermore, the tools were tested on a synthetic dataset, which was useful for testing functionality, but does not necessarily represent the condition of “real” health data, which may include numerous additional or unexpected errors and anomalies. Ideally, the team would have been able to test the tools on real patient data, but information governance approvals were not possible in the available time and a fully standardised dataset was required to ensure objectivity when comparing tools, hence a controlled synthetic dataset was most appropriate for the present purposes. While some of the tools were tested on real datasets by volunteers (Cystic Fibrosis Trust and Neonatal Data Analysis Unit), this was designed to review the initial views regarding usability of the tool, rather than provide a comparison of the outputs.

For peer review only - http://bmjopen.bmj.com/site/about/guidelines.xhtml

Determining data quality is a complex process and far harder than commonly assumed, especially for high dimensional and longitudinal data such as health data. Data profiling provides the user with an understanding of the inherent technical data quality according to various dimensions within a given dataset but does not, in itself, improve quality. Rather, based on the outcome of data profiling, it will likely be required to utilize one or more data quality tools to remediate issues detected, this being best accomplished by data analysts and/or scientists with subject matter expertise, working close to the original source of the data.

Technical data quality metrics across the dimensions described here represents only one component of overall usefulness, or utility, of a dataset. Other factors, such as source, provenance, time period, geographical coverage, etc may determine the utility for a particular project, independent of any technical data quality metrics.[11] Furthermore, data in a given data set may have an acceptable level of quality for some contexts or use cases, for example a student technical project, but the same data may be inadequate in other contexts, such as use for healthcare regulatory purposes, based on a range of factors. The concept of overall evaluation of dataset utility for specific use cases is becoming more widely recognised, for example both through data utility matrix framework development at HDRUK, and registry quality evaluation tools at NICE.[11][12]

Uptake of routine profiling of data is not yet commonplace within the health data sector, and the wider adoption of data profiling tools would encourage greater literacy and higher expectations among users of health data. Transparency of current dataset profiles, for example on the Innovation Gateway, would provide an incentive for focused improvement of data, as well as informed decision-making by users. Further work could be done in the presentation of the outputs of data profiling exercises, in order to ascertain the approach that is most conducive to effective data curation.

For peer review only - http://bmjopen.bmj.com/site/about/guidelines.xhtml

For peer review only

Evaluation of a wide range of freely available software tools for data engineering with a focus on data profiling for health care data tested using synthetic datasets has determined that several tools perform highly in a range of tasks appropriate to this use case. By the more widespread use of routine health dataset profiling, and associated remediation, along with other measures to understand and improve dataset utility, we anticipate that the overall quality of health data for research use can be increased.

FUNDING STATEMENT

This work was supported by Medical Research Council capital funding (August 2019).

CONTRIBUTORSHIP STATEMENT

BG, SV and NS conceived the study. EM, TH, OD, RJ and VR developed the methodology further, evaluated the tools and provided the initial results. KE and VB tested the tools on their own datasets and provided feedback on results. NS, BG, CF and JB prepared and drafted the manuscript. The guarantor of the content is NS.

COMPETING INTERESTS

None declared.

DATA AVAILABILITY STATEMENT

Data are available upon reasonable request.

For peer review only

For peer review only - http://bmjopen.bmj.com/site/about/guidelines.xhtml

REFERENCES

1 Home. HDR UK, https://www.hdruk.ac.uk/ (accessed 2020 August 14).

2 HDRUK Innovation Gateway | Homepage. HDR UK, https://www.healthdatagateway.org/ (accessed 12 October 2020).

3 What is Data Quality?. DAMA, https://www.dama.org/content/what-data-quality (accessed 14 August 2020).

4 Naumann F. Data profiling revisited. *ACM SIGMOD Record* 2014; 42(4):40–9.doi:10.1145/2590989.2590995.

5 Mahanti R. Critical Success Factors for Implementing Data Profiling: The First Step Toward Data Quality. *Software Quality Professional Magazine* 2014; 16(2):13-26.

6 Abedjan Z, Golab L, Naumann F. Profiling relational data: a survey. *The VLDB Journal* 2015; 24(4):557–81.doi:10.1007/s00778-015-0389-y.

7 Magic Quadrant Research Methodology. Gartner, https://www.gartner.com/en/research/methodologies/magic-quadrants-research (2019, accessed 12 October 2020).

8 Synthetic Patient Population Simulator. GitHub, https://github.com/synthetichealth/synthea (accessed 16 October 2020).

9 Critical Capabilities for Data Quality Tools. Gartner, https://www.gartner.com/en/documents/3913549 (accessed 25 February 2021).

10 DAMA Quality Dimensions. DAMA, https://www.whitepapers.em360tech.com/wp-content/files_mf/1407250286DAMAUKDQDimensionsWhitePaperR37.pdf (accessed 14 August 2020).

11 Data Utility Evaluation. HDR UK, https://www.hdruk.ac.uk/helping-with-health-

For peer review only - http://bmjopen.bmj.com/site/about/guidelines.xhtml

data/ways-to-improve-data-quality/data-utility-evaluation/ (accessed 16 April 2021).

12 REQueST Tool and its vision paper. EUnetHTA, https://eunethta.eu/request-tool-and-its-vision-paper/ (accessed 12 October 2020).

For peer review only

For peer review only - http://bmjopen.bmj.com/site/about/guidelines.xhtml

Add Tool Delete Tool	Data Ingestion and Integration	Data Ingestion and Integration	Data Ingestion and Integration	Data Ingestion and Integration	Data Preparation and Cleaning	Data Preparation and Cleaning	Data Preparation and Cleaning	Data Preparation and Cleaning	Data Preparation and Cleaning	Data Profiling, Exploration / Pattern Detection	Data Monitoring	Data Use	Data Use	Data Use	Data Use
	Connectivity	Parsing	Issue resolution and workflow	Architecture and integration	Master Reference Data Management	Standardization and cleansing	Matching, linking and merging	Address validation / geocoding	Data curation and enrichment	Data profiling, measurement and visualization	Monitoring	Metadata management	Usability	DevOps environment	Deployment environment
Knime	0.29	1.00	1.00	0.75	0.00	1.00	1.00	1.00	1.00	1.00	1.00	0.43	0.67	0.00	0.00
Pandas Profiling	0.29	0.00	0.00	0.00	0.00	0.00	0.00	0.00	0.00	1.00	0.00	0.00	0.33	0.00	0.00
Orange	0.29	1.00	1.00	0.25	0.00	0.50	1.00	1.00	0.67	1.00	1.00	0.00	0.67	0.00	0.00
RapidMiner	0.29	1.00	0.50	0.50	0.00	0.50	1.00	0.00	0.33	1.00	1.00	0.00	0.67	0.00	0.00
WEKA	0.18	0.00	0.00	0.00	0.00	0.25	0.80	0.00	0.67	1.00	0.00	0.43	0.17	0.00	0.00
Anonimatron	0.29	0.00	0.00	0.00	0.00	0.00	0.00	0.00	0.00	0.00	0.00	0.00	0.17	0.00	0.00
ARX Data Anonymization	0.29	0.00	0.00	0.00	0.00	0.25	0.00	0.00	0.33	0.00	0.00	0.00	0.33	0.00	0.00
WhiteRabbit	0.59	0.00	0.50	0.25	0.00	0.00	0.00	0.00	0.00	0.11	0.33	0.00	0.33	0.00	0.00
Aggregate Profiler (AP)	0.29	0.00	0.00	0.00	0.00	0.00	0.60	1.00	0.67	0.78	1.00	0.43	0.17	0.00	0.00
Talend Open Studio for Data Integration	0.29	1.00	0.50	0.25	0.00	0.00	0.00	0.00	0.00	0.00	0.00	0.00	0.00	0.00	0.00
Talend Open Studio For Big Data	0.29	1.00	0.50	0.25	0.00	0.00	0.00	0.00	0.00	0.00	0.00	0.00	0.00	0.00	0.00
Talend Open Studio For Data Quality	0.29	1.00	0.00	0.00	0.00	0.25	0.40	0.00	0.67	0.56	0.00	0.00	0.00	0.00	0.00
Talend Open Studio For ESB	0.29	0.00	1.00	0.00	0.00	0.00	0.00	0.00	0.00	0.00	0.00	0.00	0.00	0.00	1.00
Talend Open Studio For MDM	0.29	0.00	0.00	0.00	0.40	0.25	0.00	0.00	0.33	0.00	0.00	0.00	0.17	0.00	0.00
OpenRefine	0.18	1.00	0.00	0.25	0.00	0.25	0.80	0.00	0.00	0.00	0.00	0.00	0.00	0.00	0.00
DataCleaner	0.29	1.00	1.00	0.50	0.00	1.00	1.00	1.00	1.00	1.00	1.00	0.00	0.33	0.00	0.00
DataPreparator	0.18	0.00	0.00	0.25	0.00	0.25	0.60	0.00	0.00	0.00	0.00	0.00	0.00	0.00	0.00
Data Match	0.29	0.00	0.00	0.00	0.00	0.00	0.40	0.00	0.00	0.00	0.00	0.00	0.00	0.00	0.00
DataMartist	0.29	1.00	0.00	0.00	0.00	0.25	0.20	0.00	0.00	0.11	0.00	0.00	0.17	0.00	0.00
Pentaho Kettle	0.29	1.00	0.00	0.00	0.00	0.00	0.00	0.00	0.00	0.00	0.00	0.00	0.00	0.00	0.00
SQL Power Architect	0.29	0.00	0.00	0.25	0.00	0.00	0.00	0.00	0.00	0.00	0.00	0.14	0.00	0.00	0.00
SQL Power DQguru	0.29	0.00	0.00	0.00	0.00	0.50	0.60	1.00	0.00	0.00	0.00	0.00	0.00	0.00	0.00
DQ Analyzer	0.29	0.00	0.00	0.00	0.00	0.00	0.00	0.00	0.00	1.00	0.00	0.00	0.00	0.00	0.00
Pimcore	0.00	0.00	0.00	0.00	1.00	0.25	0.00	0.00	0.00	0.00	0.00	0.00	0.00	0.00	0.00
CytoScape	0.00	0.00	0.00	0.25	0.00	0.00	0.00	0.00	0.00	0.00	0.00	0.14	0.50	0.00	0.00
Anaconda	0.29	0.00	0.00	0.25	0.00	0.00	0.00	0.00	0.33	1.00	1.00	0.00	0.50	0.00	0.00
pyxplorer	0.24	0.00	0.00	0.00	0.00	0.00	0.00	0.00	0.00	0.11	0.00	0.00	0.00	0.00	0.00
MobyDQ	0.29	0.00	0.50	0.00	0.00	0.00	0.00	0.00	0.00	0.11	0.67	0.00	0.00	0.00	0.00

Main results of documentation based functionality for data quality categories by tool

581x311mm (57 x 57 DPI)

Figure 2. Results of profiling tasks using synthetic datasets. KNIME and Pandas performed best for overall data profiling tasks for this healthcare dataset

**0 = Not applicable**<br>**1 = Poor: most or all defined requirements not achieved**<br>**2 = Fair: some requirements not achieved**	**3 = Good: meets requirements**<br>**4 = Excellent: meets or exceeds some requirements**<br>**5 = Outstanding: significantly exceeds requirements**

Measure (key elements)	White Rabbit	Orange	Knime	WEKA	Aggregate Profiler	Data Cleaner	Pandas (Python)	Talend Open Studio - Data Quality
**COMPLETENESS - The proportion of stored data against the potential of "100% complete"**								
Percentage of requisite information available	2	4	4	3	2	3	5	1
Percent of missing data values (null / empty string)	2	4	4	4	3	3	5	1
Row counts	4	5	4	4	4	3	5	2
Highest and lowest value of key elements	0	3	5	0	0	3	5	1
Number of data values in an unusable state	0	2	2	0	0	3	5	0
**UNIQUENESS - No thing will be recorded more than once based upon how that thing is identified.**								
(Number of things in the real world) - Number of incorrect spellings etc. of same data in an element e.g. address (duplicate values)	0	2	2	0	1	2	5	2
(Number of recodes describing different things) Number of data items in adherence to expected/described data element value (distinct values at ID level)	0	1	2	0	1	2	5	1
(Number of things in real world i.e. duplicates)/(Number of records describing different things i.e. distinct records)	0	3	4	4	1	2	5	1
**TIMELINESS - The degree to which data represent reality from the required point in time.**								
Difference between Lowest date value and Highest Date Value	0	2	4	0	1	2	3	1
Number of records per month	0	1	3	0	0	2	3	0
**VALIDITY - Data are valid if it conforms to the syntax (format, type, range) of its definition.**								
Percentage of data values that comply with the specified formats (data types, ranges etc.)	0	1	3	0	0	4	5	2
Percentage of data values that don't comply to specified formats	0	0	1	0	0	1	4	0
Number of Missing values indicated e.g. with fill values	0	4	4	0	4	3	5	2
Number of Values in Specified Range	0	0	3	0	0	3	4	0
Number of values not in Specified Range	0	0	2	0	0	3	3	0
**ACCURACY - The degree to which data correctly describes the "real world" object or event being described.**								
Number of accurate data values	0	3	3	0	2	0	5	2
Number of inaccurate data values	0	0	0	0	0	0	5	0
Actual data value count versus predicted data value count	0	0	0	0	0	0	3	0
Number of rows and columns against expectations	0	0	0	0	0	0	3	0
Number of duplicates at ID level	0	4	4	4	3	3	5	3
Number of blank columns, large % of blank data, high % of same data	0	3	4	0	2	0	5	2
Distribution across various segments	0	3	0	0	0	0	5	0
Outliers on key variables	0	3	2	0	0	0	4	0
((Count of accurate objects)/ (Count of accurate objects + Counts of inaccurate objects)	0	1	1	0	0	0	3	0
**CONSISTENCY - The absence of difference, when comparing two or more representations of a thing against a definition.**								
Analysis of pattern and/or value frequency	0	0	0	0	0	0	5	0
**TOTAL SCORES**	8	49	61	19	24	42	110	21

Supplemental Material 1. List of specific tools evaluated

Tool	Connectivity	Data Sources / File Formats
**Knime** **(Data analytics, profiling, reporting and integration platform)**	Connectivity to > 5 data sources	Simple text formats (CSV, PDF, XLS, JSON, XML, etc.)
		Unstructured data types (images, documents, networks, molecules, etc.)
		Time series data
		Connect to a host of databases and data warehouses to integrate data from Oracle, Microsoft SQL, Apache Hive, and more
		Load Avro, Parquet, or ORC files from HDFS, S3, or Azure
		Access and retrieve data from sources such as Twitter, AWS S3, Google Sheets, and Azure and extended via pandas
**Pandas Profiling (using Pandas I/O)** **(Python module for exploratory data analysis (EDA))**	Connectivity to > 5 data sources	Text: - CSV, fixed-width test files, JSON, HTML, Clipboard, Excel
		Binary: OpenDocument, HDF5 Format, Feather Format, Parqeuet Format, ORC Format, Msgpak, Stata, SAS, SPSS, Python Pickle Format
		SQL, Google BigQuery
**Orange** **(Data visualization, machine learning, data profiling and mining toolkit)**	Connectivity to > 5 data sources	Excel (.xlsx), simple tab-delimited (.txt), comma-separated files (.csv) or Google Sheets document
		distance matrix: Distance File
		predictive model: Load Model
		network: Network File from Network add-on
		images: Import Images from Image Analytics add-on
		several spectroscopy files: Multifile from Spectroscopy add-on
		PostgreSQL, SQL, online repository, and extended via pandas
**RapidMiner (LIMITED FREE VERSION)** **(Integrated environment for data preparation, machine learning, deep learning, text mining, and predictive analytics)**	Connectivity to > 5 data sources	Files: CSV, Stata, Hyper (Tableau), XLS, XML, QLikView, and more
		SQL: AccessDB, HSQLDB, Microsoft SQL Server (JTDS / Microsoft), MySQL, Oracle, PostgreSQL, Sybase
		NoSQL: Cassandra, MongoDB, Solr, Splunk (read only)
		Cloud services: Amazon S3, Azure blog and data lake, Dropbox, Google, Salesforce, Twitter, Zapier, Salesforce
**WEKA** **(Machine learning**	Connectivity to < 3 data sources	Arff, JSON, CSV, xrff, dat, data, names, and more
		Database using ODBC

For peer review only

**software to solve data mining problems)**		
**Anonimatron**<br><br>**(Pseudonymizes datasets)**	Connectivity to > 5 data sources	Oracle, PostgreSQL, MySQL, DB2, MsSQL, Cloudscape, Pointbase, Firebird, IDS, Informix, Enhydra, Interbase, Hypersonic, jTurbo, SQLServer and Sybase
**ARX Data Anonymization**<br><br>**(Scalable Data Anonymization Tool - supports multiple privacy models)**	Connectivity to > 5 data sources	CSV files, MS Excel spreadsheets<br>Relational database systems, such as MS SQL, DB2, MySQL or PostgreSQL
**WhiteRabbit**<br><br>**(Tool to help prepare for ETLs of healthcare datasets)**	Connectivity to > 5 data sources	comma-separated text files<br>MySQL, SQL Server, Oracle, PostgreSQL, Microsoft APS, Microsoft Access, Amazon RedShift, Google BigQuery
**Aggregate Profiler (AP)**<br><br>**(Data profiling and analysis tool)**	Connectivity to > 5 data sources	XML, XLS or CSV format, PDF export<br>Teiid, Mysql, Oracle, Postgres, Access, Db2, SQL Server certified Big data support - HIVE
**Talend Open Studio for Data Integration (LIMITED FREE VERSION)**<br><br>**(Data integration and ETL)**	Connectivity to > 5 data sources	More than 900 pre-built connectors and components for Oracle, Teradata, Microsoft SQL server, Marketo, Salesforce, NetSuite, SAP, Microsoft Dynamics, Sugar CRM, Dropbox, Box, SMTP, FTP/SFTP, LDAP, and more
**Talend Open Studio for Big Data (LIMITED FREE**	Connectivity to > 5 data sources	Cloud: Amazon Web Services (AWS), Microsoft Azure, Google Cloud Platform, and more<br>RDBMS: Oracle, Teradata, Microsoft SQL server, and more<br>SaaS: Marketo, Salesforce, NetSuite, and more

For peer review only

**VERSION)**<br><br>**(ETL for large and diverse data sets)**		Packaged Apps: SAP, Microsoft Dynamics, Sugar CRM, and more
		Technologies: Dropbox, Box, SMTP, FTP/SFTP, LDAP, and more
**Talend Open Studio for Data Quality (LIMITED FREE VERSION)**<br><br>**(Assesses accuracy and integrity of data - Data Profiling Tool)**	Connectivity to > 5 data sources	Local or remote file that can be imported into the Talend Data Preparation tool (or from a database connection or other data sources, although not in the context of the Free Desktop version).
		Excel or CSV file
		90+ data sources and scale with Stitch Data Loader - https://www.talend.com/products/pricing-model/
**Talend Open Studio for ESB (LIMITED FREE VERSION)**	Connectivity to > 5 data sources	Cloud: Amazon Web Services (AWS), Microsoft Azure, Google Cloud Platform, and more
		RDBMS: Oracle, Teradata, Microsoft SQL server, and more
		SaaS: Marketo, Salesforce, NetSuite, and more
		Packaged Apps: SAP, Microsoft Dynamics, Sugar CRM, and more
		Technologies: Dropbox, Box, SMTP, FTP/SFTP, LDAP, and more
**Talend Open Studio for MDM (LIMITED FREE VERSION)**<br><br>**(key capabilities for data governance and master data management)**	Connectivity to > 5 data sources	AWS, Microsoft Azure, Google Cloud Platform, and more. Plus, SaaS, packaged apps, and web services
**OpenRefine**<br><br>**(Tool for cleaning and transforming data)**	Connectivity to < 3 data sources	TSV, CSV, *SV, .xls, .xlsx, JSON, XML, RDF as XML and google documents
**DataCleaner (COMMUNITY EDITION - Limited)**	Connectivity to > 5 data sources	CSV files, Excel spreadsheets
		JDBC, MySQL, PostrgreSQL, SQL Server
		Salesforce, SugarCRM

For peer review only

**(Data profiling, data cleaning, and data integration tool) - offers integration with Pentaho**		
**DataPreparator**<br><br>**(Preprocessing - data cleaning, transformation, and exploration)**	Connectivity to < 3 data sources	JDBC, XLS<br>ARFF, DATA, CSV or plain text file format
**Data Match (30-DAY FREE TRIAL)**<br><br>**(visual data cleansing application - a component of Data Ladder)**	Connectivity to > 5 data sources	Access, Apache HBase, Dynamics CRM, Email, Excel, Facebook, JSON, MongoDB, MySQL, Salesforce, SugarCRM, Twitter, XML
**DataMartist (30 DAY FREE TRIAL, STANDARD - $349, PROFESSIONAL - $995)**<br><br>**(Visual, data profiling and data transformation tool)**	Connectivity to > 5 data sources	SQL Server, Oracle, MySQL, ODBC, MS Access, Excel Spreadsheets, Delimited text files including CSV data
**Pentaho Kettle (COMMUNITY EDITION - Limited)**<br><br>**(ETL Tool) Integrates with**	Connectivity to > 5 data sources	Oracle, PostgreSQL, Redshift, SAP, SQLite, SparkSQL, Sybase, Teradata, UniVerse, Verica, Cloudera Impala, Hypersonic, H2 and more

For peer review only

**WEKA (Data Profiling)**		
**SQL Power Architect (COMMUNITY EDITION - Limited)**<br><br>**(Data Modeling & Profiling Tool)**	Connectivity to > 5 data sources	JDBC, PostgreSQL, SQL, MySQL, HSSQLDB, Oracle, DB2, HSQLDB, SQLstream, H2, Derby
**SQL Power DqGuru (COMMUNITY EDITION - Limited)**<br><br>**(Data Cleansing & MDM Tool)**	Connectivity to > 5 data sources	JDBC, Oracle, Postgress, MySQL, Sybase and more
**DQ Analyzer (COMMUNITY EDITION - Limited) (Data profiling tool)**	Connectivity to > 5 data sources	Oracle, MS SQL, DB2, Sybase, Teradata, MySQL, Apache Derby, PostgreSQL<br>CSV, TXT, and XLS(X)
**Pimcore**<br><br>**(Data Management, Integration, PIM, MDM, DAM)**	*Unable to collect during study*	*Unable to collect during study*
**CytoScape**<br><br>**(software platform for visualizing molecular interaction networks and biological pathways)**	*Unable to collect during study*	Simple interaction file (SIF or .sif format), Graph Markup Language (GML or .gml format), XGMML (extensible graph markup and modelling language), SBML, BioPAX, PSI-MI Level 1 and 2.5, Delimited text, Excel Workbook (.xls)
**Anaconda**<br><br>**(data science platform)**	Connectivity to > 5 data sources	Multiple Python Connectors

For peer review only

**Pyxplorer** (a simple tool that allows interactive profiling of datasets)	Connectivity to < 5 data sources	Hive, Impala, MySQL
**MobyDQ** (Testing tool - aims to automate Data Quality checks during data processing)	Connectivity to > 5 data sources	Cloudera Hive, MariaDB, Microsoft SQL Server, MySQL, Oracle, PostgreSQL, SQLite, Teradata, Snowflake, Hortonworks Hive

For peer review only

Supplemental Material 2. A Data profiling report produced by Pandas Profiling (Python).

Overview

Overview   Reproduction   Warnings 32

Dataset statistics

Number of variables	21
Number of observations	10
Missing cells	5
Missing cells (%)	2.4%
Duplicate rows	0
Duplicate rows (%)	0.0%
Total size in memory	1.8 KiB
Average record size in memory	180.8 B

Variable types

CAT	14
NUM	7

CITY
Categorical

HIGH CORRELATION
MISSING

Distinct count	7
Unique (%)	77.8%
Missing	1
Missing (%)	10.0%
Memory size	80.0 B

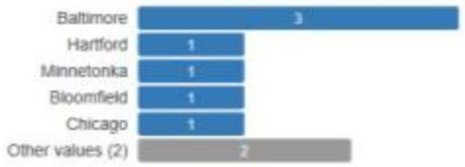

Toggle details

Common Values   Length

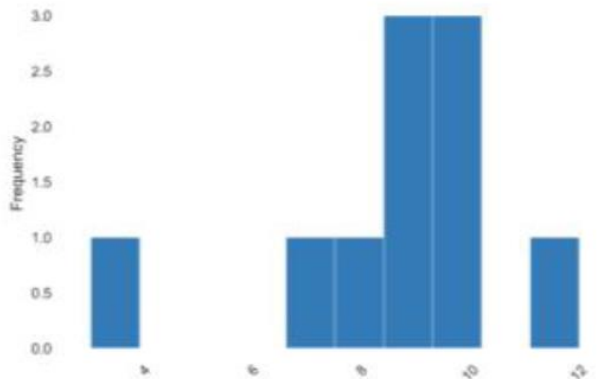

Length

Max length	12
Median length	9
Mean length	8.7
Min length	3

REVENUE
Real number ($\mathbb{R}_{\geq 0}$)
UNIQUE
ZEROS

Distinct count	10
Unique (%)	100.0%
Missing	0
Missing (%)	0.0%
Infinite	0
Infinite (%)	0.0%

Mean	87811405.0
Minimum	0.0
Maximum	154184100.0
Zeros	1
Zeros (%)	10.0%
Memory size	80.0 B

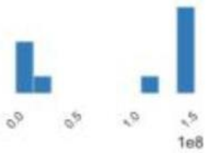

Toggle details

Statistics | Histogram(s) | Common values | Extreme values

Quantile statistics

Minimum	0
5-th percentile	587250
Q1	10433062.5
median	129576100
Q3	142068150
95-th percentile	153313215
Maximum	154184100
Range	154184100
Interquartile range (IQR)	131635087.5

Descriptive statistics

Standard deviation	70229707.73
Coefficient of variation (CV)	0.7997788867
Kurtosis	-2.177497116
Mean	87811405
Median Absolute Deviation (MAD)	23640350
Skewness	-0.4427638806
Sum	878114050
Variance	4.932211848e+15

Correlations

Pearson's r | Spearman's ρ | Kendall's τ | Phik (φk) | Cramér's V (φc)

Toggle correlation descriptions

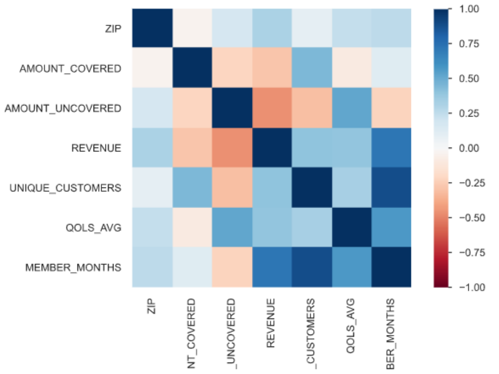

Pearson's r

The Pearson's correlation coefficient (*r*) is a measure of linear correlation between two variables. It's value lies between -1 and +1, -1 indicating total negative linear correlation, 0 indicating no linear correlation and 1 indicating total positive linear correlation. Furthermore, *r* is invariant under separate changes in location and scale of the two variables, implying that for a linear function the angle to the x-axis does not affect *r*.

To calculate *r* for two variables *X* and *Y*, one divides the covariance of *X* and *Y* by the product of their standard deviations.

BMJ Open

Evaluation of Freely Available Data Profiling Tools for Health Data Research Application: a functional evaluation review

Journal:	*BMJ Open*
Manuscript ID	bmjopen-2021-054186.R1
Article Type:	Original research
Date Submitted by the Author:	13-Feb-2022
Complete List of Authors:	Gordon, Ben; Health Data Research UK<br>Fennessy, Clara; Health Data Research UK<br>Varma, Susheel; Health Data Research UK<br>Barrett, Jake; Health Data Research UK<br>McCondochie, Enez; Inspirata Ltd<br>Heritage, Trevor; Inspirata Ltd<br>Duroe, Oenone; Inspirata Ltd<br>Jeffery, Richard; Inspirata Ltd<br>Rajamani, Vishnu; Inspirata Ltd<br>Earlam, Kieran; Cystic Fibrosis Trust<br>Banda, Victor; Imperial College London Neonatal Medicine Research Group, Neonatal Data Analysis Unit<br>Sebire, Neil; Health Data Research UK
**Primary Subject Heading**:	Health informatics
Secondary Subject Heading:	Health services research, Health policy
Keywords:	Information management < BIOTECHNOLOGY & BIOINFORMATICS, Health informatics < BIOTECHNOLOGY & BIOINFORMATICS, Information technology < BIOTECHNOLOGY & BIOINFORMATICS

SCHOLARONE™
Manuscripts

I, the Submitting Author has the right to grant and does grant on behalf of all authors of the Work (as defined in the below author licence), an exclusive licence and/or a non-exclusive licence for contributions from authors who are: i) UK Crown employees; ii) where BMJ has agreed a CC-BY licence shall apply, and/or iii) in accordance with the terms applicable for US Federal Government officers or employees acting as part of their official duties; on a worldwide, perpetual, irrevocable, royalty-free basis to BMJ Publishing Group Ltd ("BMJ") its licensees and where the relevant Journal is co-owned by BMJ to the co-owners of the Journal, to publish the Work in this journal and any other BMJ products and to exploit all rights, as set out in our licence.

The Submitting Author accepts and understands that any supply made under these terms is made by BMJ to the Submitting Author unless you are acting as an employee on behalf of your employer or a postgraduate student of an affiliated institution which is paying any applicable article publishing charge ("APC") for Open Access articles. Where the Submitting Author wishes to make the Work available on an Open Access basis (and intends to pay the relevant APC), the terms of reuse of such Open Access shall be governed by a Creative Commons licence – details of these licences and which Creative Commons licence will apply to this Work are set out in our licence referred to above.

Other than as permitted in any relevant BMJ Author's Self Archiving Policies, I confirm this Work has not been accepted for publication elsewhere, is not being considered for publication elsewhere and does not duplicate material already published. I confirm all authors consent to publication of this Work and authorise the granting of this licence.

For peer review only

Evaluation of Freely Available Data Profiling Tools for Health Data Research Application: a functional evaluation review

BEN GORDON[1], CLARA FENNESSY[1], SUSHEEL VARMA[1], JAKE BARRETT[1], ENEZ MCCONDOCHIE[2], TREVOR HERITAGE[2], OENONE DUROE[2], RICHARD JEFFERY[2], VISHNU RAJAMANI[2], KIERAN EARLAM[3], VICTOR BANDA[4], NEIL J SEBIRE[1]

1. Health Data Research UK, London, UK
2. Inspirata Ltd, Tampa, Florida, USA
3. Cystic Fibrosis Trust, London, UK
4. Neonatal Data Analysis Unit, Imperial College London, London, UK

Correspondence:

PROFESSOR NEIL J SEBIRE

Chief Clinical Data Officer, Health Data Research UK

Wellcome Trust, Gibbs Building, 215 Euston Road, London, NW1 2BE

Email: neil.sebire@hdruk.ac.uk

Word Count: 2744

For peer review only

ABSTRACT

Objectives: To objectively evaluate freely available data profiling software tools using healthcare data.

Design: Data profiling tools were evaluated for their capabilities using publicly available information and data sheets. From initial assessment, several underwent further detailed evaluation for application on healthcare data using a synthetic dataset of 1000 patients and associated data using a common health data model, and tools scored based on their functionality with this dataset.

Setting: Improving the quality of healthcare data for research use is a priority. Profiling tools can assist by evaluating datasets across a range of quality dimensions. Several freely available software packages with profiling capabilities are available but healthcare organizations often have limited data engineering capability and expertise.

Participants: 28 profiling tools, eight undergoing evaluation on synthetic dataset of 1000 patients.

Results: Of 28 potential profiling tools initially identified, eight showed high potential for applicability with healthcare datasets based on available documentation, of which two performed consistently well for these purposes across multiple tasks including determination of completeness, consistency, uniqueness, validity, accuracy and provision of distribution metrics.

Conclusions: Numerous freely available profiling tools are serviceable for potential use with health datasets, of which at least two demonstrated high performance across a range of technical data quality dimensions based on testing with synthetic health dataset and common data model. The appropriate tool choice depends on factors including underlying organizational infrastructure, level of data engineering and coding expertise, but there are

For peer review only

For peer review only - http://bmjopen.bmj.com/site/about/guidelines.xhtml

freely available tools helping profile health datasets for research use and inform curation activity.

For peer review only

For peer review only - http://bmjopen.bmj.com/site/about/guidelines.xhtml

Strengths and limitations of this study

- We are not aware of any other publication reviewing open and open-source data profiling tools using this level of rigour.
- A range of freely available data profiling tools are capability mapped regarding utility for profiling health data sets.
- Use of such data profiling software tools can help improve data quality by understanding the technical dimensions of a given health data set
- There may be other potentially suitable tools in existence that were not discovered and evaluated.
- It was not always possible to find out information on individual tools from available documentation.

For peer review only

For peer review only - http://bmjopen.bmj.com/site/about/guidelines.xhtml

INTRODUCTION

Health Data Research UK's mission is to unite the UK's health data to enable discoveries that improve people's lives. [1] One aspect of this activity is the ambition to provide a consistent view on the utility of particular datasets for specific purposes through an Innovation Gateway. [2] This would allow users to understand whether a dataset is likely to meet their needs, ahead of requesting access. One important aspect of the utility of a dataset relates to the technical dimensions of data quality, [3] as the consistent use of data quality metrics can facilitate comparison between datasets and, in addition, can demonstrate areas of potential improvement for data custodians. Data quality is frequently cited as a challenge in undertaking health research, as well as for other uses of health data. [4] Commonly used data quality dimensions in health include completeness, consistency, uniqueness, validity, accuracy, and timeliness. [5]

There are a variety of approaches used for establishing the quality of health data, hindering wider use of data due to challenges in understanding and communicating the usefulness of the data. [6]In addition to domain-specific subject matter expertise, semi-automated analysis of datasets using data quality profiling software tools can assist the process, supporting increased awareness of data quality of datasets, completeness and consistency of data submissions, improved reliability, accuracy and auditability and ultimately 'better' more usable data over time. Data profiling is the process of reviewing source data, understanding the structure, content and interrelationships of elements, examining records to discover errors/issues relating to content and format, and understanding data distributions and other factors. [7] It is seen as an important step towards improving the quality and usefulness of data. [8] There are many challenges in profiling data, depending on the structure and format of the underlying data. [9]

Many software tools are available, with varied applicability and data profiling capability for healthcare data. The aims of this study were to identify and evaluate functionality and usability of existing openly available (either open source or free-to-use) data quality assessment tools for potential users across the health data research community with specific focus on data profiling capabilities.

For peer review only - http://bmjopen.bmj.com/site/about/guidelines.xhtml

Technical data quality metrics across the dimensions described above represents only a subset of overall characteristics to describe usefulness, or utility, of a dataset. Other factors, such as source, provenance, time period, geographical coverage, etc may determine the utility for a particular project, independent of any technical data quality metrics. [10] Furthermore, data in a given data set may have an acceptable level of quality for some contexts or use cases, for example a student technical project, but the same data may be inadequate in other contexts, such as use for healthcare regulatory purposes, based on a range of factors. The concept of overall evaluation of dataset utility for specific use cases is becoming more widely recognised. [11]

For peer review only

For peer review only - http://bmjopen.bmj.com/site/about/guidelines.xhtml

METHODS

Study design

In order to evaluate existing freely available data profiling tools for potential use with health datasets, a desk-based activity was performed. This first required the identification of as many tools as possible that would be available without cost, followed by an initial evaluation of the identified tools against a range of broad criteria based on publicly available information regarding the tool functionalities. Following this evaluation, tools which scored highly in the areas of most interest for profiling of health datasets were tested on a synthetic health dataset to evaluate their capability in an objective way.

Identification of tools

An initial scoping exercise was conducted to identify data profiling tools that were freely available. This included tools that were open-source and those that were proprietary but freely available (or having a functional freely available version). The tools were identified through web searches, with inclusion criteria being the absence license restrictions, cost, lack of expert level user requirements and appropriateness of functionality as relates to health data quality. This was supplemented by discussion with individuals currently working in the sector and involved in data profiling and curation. This process resulted in 28 potential tools for initial evaluation, some of which were generic tools.

Initial Evaluation

In order to evaluate the tools, a general comparison matrix was developed based on criteria used previously for evaluating data quality tools. [12] EM identified individual functions drawing from Gartner and DAMA criteria, as well as suggesting further functions, which could be categorised into functional areas and major categories. EM and TH developed an initial categorisation of functional areas and major categories, and this was refined in collaboration with BG, SV and NJS. The scoring matrix was developed as a feature tree, comprising five major categories and fourteen minor functional areas, and a maximum score allocated for each area. The 28 tools were initially compared and categorized against the matrix using information from the available product documentation and data sheets.(Table 1)

For peer review only - http://bmjopen.bmj.com/site/about/guidelines.xhtml

For peer review only

For peer review only - http://bmjopen.bmj.com/site/about/guidelines.xhtml

Table 1. Detailed Scoring Criteria per Feature

FEATURE TREE			SCORE
**Data Ingestion and Integration**	*Data Consolidation*	Connectivity to N data sources	5
		Data Extraction, Transformation and Loading (ETL) and ETL support	5
		Data modelling	5
	Data Propagation	Data flow orchestration, Enterprise Application Integration (EAI), exchange of messages and transactions	5
		Enterprise Data Replication (EDR), transfer large amounts of data between databases	5
		Versioning and file management	5
	Data Virtualization	Data access	5
	Data Federation	Enterprise Information Integration (EII)	5
		**Total**	**40**
**Data Preparation and Cleaning**	*Parsing and Standardization*	Tagging data with keywords, descriptions or categories	5
		Data scrubbing/cleansing/handling blank values/reformatting values/threshold checking	5
		Data enhancement/enrichment/curation	5
		Natural Language Processing	5
		Address validation/geocoding	5
		Master data management	5
		Data masking	5
	Identity Resolution, Linkage, Merging & Consolidation	Data de-duping	5
		Machine Learning (ML) / training a statistical model	5
		Data aggregation	5
		Data binning	5
		Grouping similar data / clustering	5
		Outlier detection and removal	5
	Master Reference Data Management	"Hub" infrastructure to source and distribute master/reference data	5
		Master data versioning based on data history and timelines	5
		Workflow integrations to steward and publish the master/reference data	5
		Graph data stores to define relationships for creating a flexible knowledge graph	5
		Accessible API for real-time access to shared reference data	5
		**Total**	**90**
**Data Profiling, Exploration/**	*Relationship discovery*	Cross table redundancy analysis	5
		Performing data quality assessment, risk of performing joins on the data	5

For peer review only

Category	Sub-category	Feature	Score
**Pattern Detection**		Identifying distributions, key candidates, foreign-key candidates, functional dependencies, embedded value dependencies, and performing inter-table analysis.	5
	Content discovery	Data pattern discovery	5
		Domain analysis	5
		Discovering metadata and assessing its accuracy	5
	Structure discovery	Column value frequency analysis & statistics, collecting descriptive statistics like min, max, count and sum.	5
		Table structure analysis, collecting data types, length and recurring patterns.	5
		Drill-through analysis	5
		**Total**	**45**
**Data Monitoring**	*Monitoring & Alerting*	Time series data identified and collection by metric name and key/value pairs	5
		Flexible query language to leverage this dimensionality	5
		Graphing and dashboarding support	5
		**Total**	**15**
**Data Use**	*Metadata Management*	Concept identification and naming	5
		Data categorization	5
		Lineage	5
		Relationship with other metadata	5
		Comments and remarks	5
		Data statistics (profiles)	5
		Knowledge graph	5
	Privacy & Security	Data anonymization	5
		Role based access control	5
		Secure environment setup and deployment	5
		Container based deployment	5
	Data Mining	Interactive data visualization	5
		Visual programming and analysis	5
		Visual illustrations & training documentation	5
		Sample data / generate fake data	5
		Add-ons and extension functionality	5
		**Total**	**80**

Each tool was ranked based on key capabilities required to address the profiling aspects of data quality using the feature tree and scoring. Tools were assigned the available weighted scoring based on the ability to provide the function described, according to the information available. Each feature was scored using a binary system, either 0 or 5. An exception to this

For peer review only

For peer review only - http://bmjopen.bmj.com/site/about/guidelines.xhtml

rule is the "Connectivity to N data sources" where this feature is scored 3, 4, and 5 when a tool has connectivity to < 3, < 6, and > 5 data sources, respectively. Scores for each of the five major category areas were converted to a percentage of the total available score for that area.

In-depth evaluation

Following the initial evaluation, eight tools scored were selected for further, in-depth evaluation based on the data profiling major category score and functions (the focus of this process was to evaluate data profiling capabilities; other potential functionalities were recorded for interest as above but not used for ranking). The selected tools included: Knime, DataCleaner, Orange, WEKA, Pandas-profiling (Python), Aggregate Profiler, Talend Open Studio for Data Quality, WhiteRabbit. (Rapid Miner and DQ Analyzer were excluded since they were limited free versions of paid-for tools. Since two python tools, Pandas Profiling and Anaconda, scored highly for profiling, only Pandas profiling was further evaluated since it is explicitly intended for data profiling. Finally, WhiteRabbit, Talend Open Studio for Data Quality and Aggregate Profiler were also evaluated since they were identified as being used by the HDR UK community). To evaluate these tools for their data profiling performance and capability, synthetic data sets were created using the open source tool, Synthea to generate CSV files and SQL Database adhering to the Observational Medical Outcomes Partnership Common Data Model (an internationally adopted data standard) containing 1000 patients and related clinical data and the tools run on this dataset. [13]Synthea allows generation of fully synthetic datasets which broadly conform to the data types and values expected in a 'real' health dataset but with no risk of patient data identification. [14] To evaluate performance and scalability of each tool an additional synthetic dataset of 1.3 million records was also generated.

Each of the shortlisted open-source data profiling tools were evaluated based on how possible it was to execute common specific profiling functions as described in the tool documentation decided based on the Gartner reports. [15]

Further to the initial evaluation, the shortlisted tools were evaluated in-depth based on the ability to deliver data profiles against core DAMA UK data quality dimensions, [3] including

For peer review only - http://bmjopen.bmj.com/site/about/guidelines.xhtml

completeness (the proportion of stored data against the potential of 100% complete), consistency (the absence of difference, when comparing two or more representations of a thing against a definition), uniqueness (nothing recorded more than once based upon how that thing is identified), validity (data are valid if it conforms to the syntax (format, type, range) of its definition), accuracy (the degree to which data correctly describes the object or event being described) and timeliness (the degree to which data represent reality from the required point in time). For each data profiling functionality, tools were run and subjectively scored on a scale of 0-5 according to a semi-structured scale (0=unable to process, 1=most requirements not achieved, 2=some requirements not achieved, 3=meets core requirements, 4=meets and exceeds some requirements, 5=significantly exceeds core requirements).

The suitability of the tools for potential future use by other parties was estimated based on feedback from volunteers from the HDR UK community testing selected tools on their local datasets and providing a qualitative comment on usability. Formal evaluation of the tools of a range of real-world health datasets in a range of environments was outside the scope of this study.

Patient and Public Involvement

Patients and/or the public were not involved in the design, or conduct, or reporting, or dissemination plans of this research.

For peer review only

For peer review only - http://bmjopen.bmj.com/site/about/guidelines.xhtml

RESULTS

Initial evaluation

The initial 28 tools evaluated are shown in Online Supplemental Material 1 along with scores in the various data quality task categories with detailed results for data profiling functionality. The overall results of the initial scoring are shown in Figure 1, where scores have been normalised to a maximum of 1 to support initial inspection.

Subsequent evaluation

Based on the in-depth review of the selected eight tools to evaluate their ability to deliver key functions, the Python library, Pandas Profiling, was identified as possessing the most versatile functionality, able to complete all 30 of the identified profiling functions on the synthetic dataset for testing. The next most versatile tool, Knime, was able to perform 19 such tasks. Across the functionality types, Single Column – Cardinalities was one that the most tools were capable of delivering, with all tools able to deliver three of the functions in this type. The functionality type that was least well served by the tools was Dependencies, with only Pandas Profiling able to deliver any of these functions.(Table 2)

Table 2. Specific Data Profiling Tool Functionalities Evaluated

*** Key:**
K=Knime; DC=DataCleaner; O=Orange; W=WEKA; PP=Pandas Profiling (Python); AP=Aggregate Profiler; TOS=Talend Open Studio for Data Quality; WR=WhiteRabbit

FUNCTIONALITY TYPE	FUNCTION	DATA PROFILING TOOLS CAPABLE OF NATIVELY EXECUTING FUNCTION *							
		K	DC	O	W	PP	AP	TOS	WR
**Single Column – Cardinalities** REFERS TO THE UNIQUENESS OF DATA VALUES CONTAINED IN A PARTICULAR COLUMN (ATTRIBUTE) OF A TABLE (ENTITY)	*Number of rows*	✓	✓	✓	✓	✓	✓	✓	✓
	Number of nulls	✓	✓	✓	✓	✓	✓	✓	✓
	Percentage of nulls	✓		✓	✓	✓		✓	✓
	Number of distinct values (cardinality)	✓	✓	✓	✓	✓	✓	✓	✓
	Percentage of distinct values (Number of distinct values divided by the number of rows)	✓			✓	✓		✓	
**Single Column - Value distributions** PRESENTS AN ORDERING OF THE RELATIVE FREQUENCY (COUNT	*Frequency histograms (equi-width, equi-depth, etc.)*	✓				✓			
	Minimum and maximum values in a numeric column	✓	✓	✓		✓	✓	✓	✓

For peer review only - http://bmjopen.bmj.com/site/about/guidelines.xhtml

and percentage) of the assignment of distinct values	*Constancy (Frequency of most frequent value divided by number of rows)*	✓				✓		✓	
	Quartiles (3 points that divide the numeric values into 4 equal groups)	✓	✓			✓	✓	✓	✓
	Distribution of first digit in numeric values (to check Benford's law)	✓				✓		✓	
**Single Column - Patterns, datatypes, and domains** Refers to the discovery of patterns and data types	*Basic types (e.g., numeric, alphanumeric, date, time)*	✓				✓			
	DBMS-specific data type (e.g., varchar, timestamp)	✓	✓			✓	✓	✓	✓
	Measurement of Value length (minimum, maximum, average, median)	✓	✓	✓		✓	✓		✓
	Maximum number of digits in numeric values	✓	✓			✓	✓		
	Maximum number of decimals in numeric values	✓				✓	✓		
	Histogram of value patterns (Aa9...)	✓	✓			✓		✓	
	Generic semantic data type (e.g., code, date/time, quantity, identifier)	✓	✓			✓		✓	
	Semantic domain (e.g., credit card, first name, city)	✓	✓			✓		✓	
**Dependencies** Determines the dependent relationships within a data set	*Unique column combinations (UCCs) (key discovery)*					✓			
	Relaxed unique column combinations					✓			
	Inclusion dependencies (INDs) (foreign key discovery)					✓			
	Relaxed inclusion dependencies					✓			
	Functional dependencies					✓			
	Conditional functional dependencies					✓			
**Advanced Multi Column profiling** Determines the similarities and differences in syntax and data types between tables (entities) to determine which data might be redundant and which could be mapped together	*Correlation analysis*			✓		✓	✓		
	Association rule mining					✓			
	Cluster analysis					✓			
	Outlier detection	✓		✓		✓			
	Exact duplicate tuple detection		✓			✓		✓	
	Relaxed duplicate tuple detection		✓			✓		✓	
	**Total**	**19**	**13**	**8**	**5**	**30**	**10**	**15**	**8**

The tools were further evaluated based on their ability to deliver data profiles against the DAMA dimensions.(Figure 2) Pandas Profiling achieved significantly greater results compared

For peer review only - http://bmjopen.bmj.com/site/about/guidelines.xhtml

to the other tools, scoring 110 of the available points, compared to the next highest tool, Knime, with 61 points. Of the tools examined, WhiteRabbit had the least comprehensive functionality in this area, able only to provide information against the Completeness element. Across the different elements, Completeness was best served by the profiling tools, with all tools able to provide some functionality in this area. The least well-served element was Consistency, with only Pandas Profiling able to provide any output for this element. Online Supplemental Material 2 shows the profile reporting information produced by Pandas Profiling with features including basic dataset statistics overview, reports on specific numerical or categorical variables, and correlations between variables.

Links for all tools tested are available here (https://github.com/HDRUK/data-utility-tools).

User testing feedback

To provide anecdotal feedback on the usability of the tools, five of the eight tools (DataCleaner, Orange, MobyDQ, Knime and Aggregate profiler) were tested by volunteers from the Cystic Fibrosis Trust and the Neonatal Medicine Research Group. These tools were selected for testing based of the volunteer's ability and the resources available to run them.

MobyDQ and Aggregate Profiler both presented difficulties to the volunteers due to challenges installing and running the software. MobyDQ failed to authenticate due to issues with private keys and Aggregate Profiler crashed upon attempts to update.

Knime, DataCleaner and Orange could be run successfully by the volunteers. Orange required the local migration of data and installation of two additional modules, and was supported more effectively on Mac OS and Linux than Windows. Knime was fairly resource intensive and initially difficult to use, but was seen to be capable of a range of functions. DataCleaner was reported to be relatively easy to set up and run, even on a Windows machine, and capable of linking to existing databases.

For peer review only - http://bmjopen.bmj.com/site/about/guidelines.xhtml

DISCUSSION

The findings of the present study have demonstrated that numerous openly available data profiling tools are available, with several able to perform well using health datasets. The precise choice of tool for organisations will depend on the data type, model and format, in addition to IT environment, such as Windows or Linux, and expertise with such tools and coding languages, such as Python. Regardless of the tools used, appropriate deployment and dataset evaluation through data profiling should lead to early detection of data quality issues for particular data sets and sources and consequent ability to remediate such issues. The identification of Pandas Profiling as a versatile approach to data profiling is reinforced by the fact that, as a Python library, it can be combined with other tools, such as Orange or Knime, to provide an even more in-depth output.

This study provides a useful resource for individuals anywhere in the world to understand the functionality of freely available data profiling tools for use with health datasets, and put these to use. The creation of an open and persistent resource is a strength of the study. All the outputs of the testing, as well as the generated dataset, are available (https://github.com/HDRUK/data-utility-tools). None of the tested tools are specific to health data, and therefore could be used in any other domain. However, the open nature of the search for the tools, the absence of an indexed repository of these tools was likely non-exhaustive. There may be additional tools that would also have been suitable for this exercise that were not identified during the project. Furthermore, the tools were tested on a synthetic dataset, which was useful for testing functionality, but does not necessarily represent the condition of "real" health data, which may include numerous additional or unexpected errors and anomalies. Ideally, the team would have been able to test the tools on real patient data, but information governance approvals were not possible in the available time and a fully standardised dataset was required to ensure objectivity when comparing tools, hence a controlled synthetic dataset was most appropriate for the present purposes. While some of the tools were tested on real datasets by volunteers (Cystic Fibrosis Trust and Neonatal Data Analysis Unit), this was designed to review the initial views regarding usability of the tool, rather than provide a comparison of the outputs.

For peer review only - http://bmjopen.bmj.com/site/about/guidelines.xhtml

Determining data quality is a complex process and far harder than commonly assumed, especially for high dimensional and longitudinal data such as health data. Data profiling provides the user with an understanding of the inherent technical data quality according to various dimensions within a given dataset but does not, in itself, improve quality. Rather, based on the outcome of data profiling, it will likely be required to utilize one or more data quality tools to remediate issues detected, this being best accomplished by data analysts and/or scientists with subject matter expertise, working close to the original source of the data. While the ability of the tools to be used by individuals with limited experience was not the focus of this research, this would be interesting to explore in future work, particularly because the tools with the broadest capability, Pandas Profiling, was not tested by volunteers.

Further research would be useful to understand the capability of the tools in handling increasingly large sets of data. While the tools were tested against a dataset of over one million patient records, processing time was not compared quantitatively. Further, in a healthcare or health research setting, it is not unusual for a dataset to be several orders of magnitude larger than this. For a tool to be useful in these settings, it should be able to process large datasets, and within a reasonable time.

As referenced in the Introduction, there is a need for greater consistency in how dimensions of data quality are assessed and communicated. The wider adoption of data profiling tools would encourage greater literacy and higher expectations among users of health data. Transparency of current dataset profiles, for example on the Innovation Gateway, would provide an incentive for focused improvement of data, as well as informed decision-making by users. Further work could be done in the presentation of the outputs of data profiling exercises, in order to ascertain the approach that is most conducive to effective data curation.

Evaluation of a wide range of freely available software tools for data engineering with a focus on data profiling for health care data tested using synthetic datasets has determined that several tools perform highly in a range of tasks appropriate to this use case. By the more widespread use of routine health dataset profiling, and associated remediation, along with

For peer review only - http://bmjopen.bmj.com/site/about/guidelines.xhtml

other measures to understand and improve dataset utility, we anticipate that the overall quality of health data for research use can be increased.

FUNDING STATEMENT

This work was supported by Medical Research Council capital funding (August 2019). There is no grant number associated with capital fund awards.

CONTRIBUTORSHIP STATEMENT

BG, SV and NS conceived the study. EM, TH, OD, RJ and VR developed the methodology further, evaluated the tools and provided the initial results. KE and VB tested the tools on their own datasets and provided feedback on results. NS, BG, CF and JB prepared and drafted the manuscript. The guarantor of the content is NS.

COMPETING INTERESTS

None declared. EM, TH, OD, RJ, VR were employed by Inspirata Ltd at the time of the work but were contracted by HDR UK to carry out this work independently on behalf of HDR UK.

ETHICS APPROVAL

As a desk-based project, involving no patients or other human subjects, having no relation to clinical protocols and not intending to provide generalisable results, no ethical approval was required.

DATA AVAILABILITY STATEMENT

Data are available upon reasonable request.

FIGURE CAPTION

Figure 1: Main results of documentation based functionality for data quality categories by tool

Figure 2: Results of profiling tasks using synthetic datasets. KNIME and Pandas performed best for overall data profiling tasks for this healthcare dataset

For peer review only - http://bmjopen.bmj.com/site/about/guidelines.xhtml

For peer review only

For peer review only - http://bmjopen.bmj.com/site/about/guidelines.xhtml

References

[1] Health Data Research UK, "Home," HDR UK, [Online]. Available: https://www.hdruk.ac.uk. [Accessed 14 August 2020].

[2] Health Data Research UK, "HDR UK Innovation Gateway," HDR UK, [Online]. Available: https://www.healthdatagateway.org/. [Accessed 12 October 2020].

[3] A. Black and P. v. Nederpelt, "Code for Information Quality 2019," 5 September 2020. [Online]. Available: http://www.dama-nl.org/wp-content/uploads/2020/09/DDQ-Dimensions-of-Data-Quality-Research-Paper-version-1.2-d.d.-3-Sept-2020.pdf. [Accessed 3 February 2022].

[4] T. Botsis, G. Hartvigsen, F. Chei and C. Weng, "Secondary Use of EHR: Data Quality Issues and Informatics Opportunities," *Summit on Translat Bioinforma,* pp. 1-5, 2010.

[5] H. Chen, D. Hailey, N. Wang and P. Yu, "A Review of Data Quality Assessment Methods for Public Health Information Systems," *Int. J. Environ. Res. Public Health,* vol. 11, no. 5, pp. 5170-5270, 2014.

[6] M. Mashoufi, H. Ayatollahi and D. Khorasani-Zavareh, "A Review of Data Quality Assessment in Emergency Medical Services," *Open Med Inform J.,* vol. 12, pp. 19-32, 2018.

[7] F. Naumann, "Data profiling revisited," *ACM SIGMOD Record,* vol. 42, no. 4, pp. 40-49, 2013.

[8] R. Mahanti, "Critical Success Factors for Implementing Data Profiling," *Software Quality Professional,* vol. 16, no. 2, pp. 13-26, 2014.

[9] Z. Abedjan, L. Golab and F. Naumann, "Profiling relational data: a survey," *The VLDB Journal volume,* vol. 24, pp. 557-581, 2015.

[10] B. Gordon, J. Barrett, C. Fennessy, C. Cake, A. Milward, C. Irwin, M. Jones and N. Sebire, "Development of a data utility framework to support effective health data curation," *BMJ Health & Care Informatics,* vol. 28, pp. e100303. doi: 10.1136/bmjhci-2020-100303, 2021.

[11] EUnetHTA, "REQueST Tool and its Vision Paper," EUnetHTA, [Online]. Available: https://www.eunethta.eu/request-tool-and-its-vision-paper/. [Accessed 22 October 2020].

[12] Gartner, "Magic Quandrant Research Methodology," Gartner, 2019. [Online]. Available: https://www.gartner.com/en/research/methodologies/magic-quadrants-research. [Accessed 12 October 2022].

[13] OHDSI (Chapter lead: Clair Blacketer), "Chapter 4 The Common Data Model | The Book of OHDSI," 11 1 2021. [Online]. Available: https://ohdsi.github.io/TheBookOfOhdsi/CommonDataModel.html. [Accessed 3 February 2022].

[14] Synthea, "GitHub - synthetichealth/synthea," GitHub, 31 January 2022. [Online]. Available: https://github.com/synthetichealth/synthea. [Accessed 3 February 2022].

For peer review only - http://bmjopen.bmj.com/site/about/guidelines.xhtml

[15] Gartner, "Critical Capabilities for Data Quality Tools," Gartner, 14 May 2019. [Online]. Available: https://www.gartner.com/en/documents/3913549. [Accessed 21 February 2021].

For peer review only

For peer review only - http://bmjopen.bmj.com/site/about/guidelines.xhtml

Add Tool Delete Tool	Data Ingestion and Integration	Data Ingestion and Integration	Data Ingestion and Integration	Data Ingestion and Integration	Data Preparation and Cleaning	Data Preparation and Cleaning	Data Preparation and Cleaning	Data Preparation and Cleaning	Data Preparation and Cleaning	Data Profiling, Exploration / Pattern Detection	Data Monitoring	Data Use	Data Use	Data Use	Data Use
	Connectivity	Parsing	Issue resolution and workflow	Architecture and integration	Master Reference Data Management	Standardization and cleansing	Matching, linking and merging	Address validation / geocoding	Data curation and enrichment	Data profiling, measurement and visualization	Monitoring	Metadata management	Usability	DevOps environment	Deployment environment
Knime	0.29	1.00	1.00	0.75	0.00	1.00	1.00	1.00	1.00	1.00	1.00	0.43	0.67	0.00	0.00
Pandas Profiling	0.29	0.00	0.00	0.00	0.00	0.00	0.00	0.00	0.00	1.00	0.00	0.00	0.33	0.00	0.00
Orange	0.29	1.00	1.00	0.25	0.00	0.50	1.00	1.00	0.67	1.00	1.00	0.00	0.67	0.00	0.00
RapidMiner	0.29	1.00	0.50	0.50	0.00	0.50	1.00	0.00	0.33	1.00	1.00	0.00	0.67	0.00	0.00
WEKA	0.18	0.00	0.00	0.00	0.00	0.25	0.80	0.00	0.67	1.00	0.00	0.43	0.17	0.00	0.00
Anonimatron	0.29	0.00	0.00	0.00	0.00	0.00	0.00	0.00	0.00	0.00	0.00	0.00	0.17	0.00	0.00
ARX Data Anonymization	0.29	0.00	0.00	0.00	0.00	0.25	0.00	0.00	0.33	0.00	0.00	0.00	0.33	0.00	0.00
WhiteRabbit	0.59	0.00	0.50	0.25	0.00	0.00	0.00	0.00	0.00	0.11	0.33	0.00	0.33	0.00	0.00
Aggregate Profiler (AP)	0.29	0.00	0.00	0.00	0.00	0.00	0.60	1.00	0.67	0.78	1.00	0.43	0.17	0.00	0.00
Talend Open Studio for Data Integration	0.29	1.00	0.50	0.25	0.00	0.00	0.00	0.00	0.00	0.00	0.00	0.00	0.00	0.00	0.00
Talend Open Studio For Big Data	0.29	1.00	0.50	0.25	0.00	0.00	0.00	0.00	0.00	0.00	0.00	0.00	0.00	0.00	0.00
Talend Open Studio For Data Quality	0.29	1.00	0.00	0.00	0.00	0.25	0.40	0.00	0.67	0.56	0.00	0.00	0.00	0.00	0.00
Talend Open Studio For ESB	0.29	0.00	1.00	0.00	0.00	0.00	0.00	0.00	0.00	0.00	0.00	0.00	0.00	0.00	1.00
Talend Open Studio For MDM	0.29	0.00	0.00	0.00	0.40	0.25	0.00	0.00	0.33	0.00	0.00	0.00	0.17	0.00	0.00
OpenRefine	0.18	1.00	0.00	0.25	0.00	0.25	0.80	0.00	0.00	0.00	0.00	0.00	0.00	0.00	0.00
DataCleaner	0.29	1.00	1.00	0.50	0.00	1.00	1.00	1.00	1.00	1.00	1.00	0.00	0.33	0.00	0.00
DataPreparator	0.18	0.00	0.00	0.25	0.00	0.25	0.60	0.00	0.00	0.00	0.00	0.00	0.00	0.00	0.00
Data Match	0.29	0.00	0.00	0.00	0.00	0.00	0.40	0.00	0.00	0.00	0.00	0.00	0.00	0.00	0.00
DataMartist	0.29	1.00	0.00	0.00	0.00	0.25	0.20	0.00	0.00	0.11	0.00	0.00	0.17	0.00	0.00
Pentaho Kettle	0.29	1.00	0.00	0.00	0.00	0.00	0.00	0.00	0.00	0.00	0.00	0.00	0.00	0.00	0.00
SQL Power Architect	0.29	0.00	0.00	0.25	0.00	0.00	0.00	0.00	0.00	0.00	0.00	0.14	0.00	0.00	0.00
SQL Power DQguru	0.29	0.00	0.00	0.00	0.00	0.50	0.60	1.00	0.00	0.00	0.00	0.00	0.00	0.00	0.00
DQ Analyzer	0.29	0.00	0.00	0.00	0.00	0.00	0.00	0.00	0.00	1.00	0.00	0.00	0.00	0.00	0.00
Pimcore	0.00	0.00	0.00	0.00	1.00	0.25	0.00	0.00	0.00	0.00	0.00	0.00	0.00	0.00	0.00
CytoScape	0.00	0.00	0.00	0.25	0.00	0.00	0.00	0.00	0.00	0.00	0.00	0.14	0.50	0.00	0.00
Anaconda	0.29	0.00	0.00	0.25	0.00	0.00	0.00	0.00	0.33	1.00	1.00	0.00	0.50	0.00	0.00
pyxplorer	0.24	0.00	0.00	0.00	0.00	0.00	0.00	0.00	0.00	0.11	0.00	0.00	0.00	0.00	0.00
MobyDQ	0.29	0.00	0.50	0.00	0.00	0.00	0.00	0.00	0.00	0.11	0.67	0.00	0.00	0.00	0.00

Main results of documentation based functionality for data quality categories by tool

581x311mm (57 x 57 DPI)

Figure 2. Results of profiling tasks using synthetic datasets. KNIME and Pandas performed best for overall data profiling tasks for this healthcare dataset

**0 = Unable to process**<br>**1 = Poor: most or all defined requirements not achieved**<br>**2 = Fair: some requirements not achieved**	**3 = Good: meets requirements**<br>**4 = Excellent: meets or exceeds some requirements**<br>**5 = Outstanding: significantly exceeds core requirements**

Measure (key elements)	White Rabbit	Orange	Knime	WEKA	Aggregate Profiler	Data Cleaner	Pandas (Python)	Talend Open Studio - Data Quality
**COMPLETENESS - The proportion of stored data against the potential of "100% complete"**								
Percentage of requisite information available	2	4	4	3	2	3	5	1
Percent of missing data values (null / empty string)	2	4	4	4	3	3	5	1
Row counts	4	5	4	4	4	3	5	2
Highest and lowest value of key elements	0	3	5	0	0	3	5	1
Number of data values in an unusable state	0	2	2	0	0	3	5	0
**UNIQUENESS - No thing will be recorded more than once based upon how that thing is identified.**								
(Number of things in the real world) - Number of incorrect spellings etc. of same data in an element e.g. address (duplicate values)	0	2	2	0	1	2	5	2
(Number of recodes describing different things) Number of data items in adherence to expected/described data element value (distinct values at ID level)	0	1	2	0	1	2	5	1
(Number of things in real world i.e. duplicates)/(Number of records describing different things i.e. distinct records)	0	3	4	4	1	2	5	1
**TIMELINESS - The degree to which data represent reality from the required point in time.**								
Difference between Lowest date value and Highest Date Value	0	2	4	0	1	2	3	1
Number of records per month	0	1	3	0	0	2	3	0
**VALIDITY - Data are valid if it conforms to the syntax (format, type, range) of its definition.**								
Percentage of data values that comply with the specified formats (data types, ranges etc.)	0	1	3	0	0	4	5	2
Percentage of data values that don't comply to specified formats	0	0	1	0	0	1	4	0
Number of Missing values indicated e.g. with fill values	0	4	4	0	4	3	5	2
Number of Values in Specified Range	0	0	3	0	0	3	4	0
Number of values not in Specified Range	0	0	2	0	0	3	3	0
**ACCURACY - The degree to which data correctly describes the "real world" object or event being described.**								
Number of accurate data values	0	3	3	0	2	0	5	2
Number of inaccurate data values	0	0	0	0	0	0	5	0
Actual data value count versus predicted data value count	0	0	0	0	0	0	3	0
Number of rows and columns against expectations	0	0	0	0	0	0	3	0
Number of duplicates at ID level	0	4	4	4	3	3	5	3
Number of blank columns, large % of blank data, high % of same data	0	3	4	0	2	0	5	2
Distribution across various segments	0	3	0	0	0	0	5	0
Outliers on key variables	0	3	2	0	0	0	4	0
((Count of accurate objects)/ (Count of accurate objects + Counts of inaccurate objects)	0	1	1	0	0	0	3	0
**CONSISTENCY - The absence of difference, when comparing two or more representations of a thing against a definition.**								
Analysis of pattern and/or value frequency	0	0	0	0	0	0	5	0
**TOTAL SCORES**	8	49	61	19	24	42	110	21

Supplemental Material 1. List of specific tools evaluated

Tool	Connectivity	Data Sources / File Formats
**Knime** **(Data analytics, profiling, reporting and integration platform)**	Connectivity to > 5 data sources	Simple text formats (CSV, PDF, XLS, JSON, XML, etc.)
		Unstructured data types (images, documents, networks, molecules, etc.)
		Time series data
		Connect to a host of databases and data warehouses to integrate data from Oracle, Microsoft SQL, Apache Hive, and more
		Load Avro, Parquet, or ORC files from HDFS, S3, or Azure
		Access and retrieve data from sources such as Twitter, AWS S3, Google Sheets, and Azure and extended via pandas
**Pandas Profiling (using Pandas I/O)** **(Python module for exploratory data analysis (EDA))**	Connectivity to > 5 data sources	Text: - CSV, fixed-width test files, JSON, HTML, Clipboard, Excel
		Binary: OpenDocument, HDF5 Format, Feather Format, Parqeuet Format, ORC Format, Msgpak, Stata, SAS, SPSS, Python Pickle Format
		SQL, Google BigQuery
**Orange** **(Data visualization, machine learning, data profiling and mining toolkit)**	Connectivity to > 5 data sources	Excel (.xlsx), simple tab-delimited (.txt), comma-separated files (.csv) or Google Sheets document
		distance matrix: Distance File
		predictive model: Load Model
		network: Network File from Network add-on
		images: Import Images from Image Analytics add-on
		several spectroscopy files: Multifile from Spectroscopy add-on
		PostgreSQL, SQL, online repository, and extended via pandas
**RapidMiner (LIMITED FREE VERSION)** **(Integrated environment for data preparation, machine learning, deep learning, text mining, and predictive analytics)**	Connectivity to > 5 data sources	Files: CSV, Stata, Hyper (Tableau), XLS, XML, QLikView, and more
		SQL: AccessDB, HSQLDB, Microsoft SQL Server (JTDS / Microsoft), MySQL, Oracle, PostgreSQL, Sybase
		NoSQL: Cassandra, MongoDB, Solr, Splunk (read only)
		Cloud services: Amazon S3, Azure blog and data lake, Dropbox, Google, Salesforce, Twitter, Zapier, Salesforce
**WEKA** **(Machine learning**	Connectivity to < 3 data sources	Arff, JSON, CSV, xrff, dat, data, names, and more
		Database using ODBC

For peer review only

**software to solve data mining problems)**		
**Anonimatron**<br><br>**(Pseudonymizes datasets)**	Connectivity to > 5 data sources	Oracle, PostgreSQL, MySQL, DB2, MsSQL, Cloudscape, Pointbase, Firebird, IDS, Informix, Enhydra, Interbase, Hypersonic, jTurbo, SQLServer and Sybase
**ARX Data Anonymization**<br><br>**(Scalable Data Anonymization Tool - supports multiple privacy models)**	Connectivity to > 5 data sources	CSV files, MS Excel spreadsheets
		Relational database systems, such as MS SQL, DB2, MySQL or PostgreSQL
**WhiteRabbit**<br><br>**(Tool to help prepare for ETLs of healthcare datasets)**	Connectivity to > 5 data sources	comma-separated text files
		MySQL, SQL Server, Oracle, PostgreSQL, Microsoft APS, Microsoft Access, Amazon RedShift, Google BigQuery
**Aggregate Profiler (AP)**<br><br>**(Data profiling and analysis tool)**	Connectivity to > 5 data sources	XML, XLS or CSV format, PDF export
		Teiid, Mysql, Oracle, Postgres, Access, Db2, SQL Server certified Big data support - HIVE
**Talend Open Studio for Data Integration (LIMITED FREE VERSION)**<br><br>**(Data integration and ETL)**	Connectivity to > 5 data sources	More than 900 pre-built connectors and components for Oracle, Teradata, Microsoft SQL server, Marketo, Salesforce, NetSuite, SAP, Microsoft Dynamics, Sugar CRM, Dropbox, Box, SMTP, FTP/SFTP, LDAP, and more
**Talend Open Studio for Big Data (LIMITED FREE VERSION)**<br><br>**(ETL for large and diverse data sets)**	Connectivity to > 5 data sources	Cloud: Amazon Web Services (AWS), Microsoft Azure, Google Cloud Platform, and more
		RDBMS: Oracle, Teradata, Microsoft SQL server, and more
		SaaS: Marketo, Salesforce, NetSuite, and more
		Packaged Apps: SAP, Microsoft Dynamics, Sugar CRM, and more
		Technologies: Dropbox, Box, SMTP, FTP/SFTP, LDAP, and more

For peer review only

**Talend Open Studio for Data Quality (LIMITED FREE VERSION)**<br><br>**(Assesses accuracy and integrity of data - Data Profiling Tool)**	Connectivity to > 5 data sources	Local or remote file that can be imported into the Talend Data Preparation tool (or from a database connection or other data sources, although not in the context of the Free Desktop version).<br>Excel or CSV file<br>90+ data sources and scale with Stitch Data Loader - https://www.talend.com/products/pricing-model/
**Talend Open Studio for ESB (LIMITED FREE VERSION)**	Connectivity to > 5 data sources	Cloud: Amazon Web Services (AWS), Microsoft Azure, Google Cloud Platform, and more<br>RDBMS: Oracle, Teradata, Microsoft SQL server, and more<br>SaaS: Marketo, Salesforce, NetSuite, and more<br>Packaged Apps: SAP, Microsoft Dynamics, Sugar CRM, and more<br>Technologies: Dropbox, Box, SMTP, FTP/SFTP, LDAP, and more
**Talend Open Studio for MDM (LIMITED FREE VERSION)**<br><br>**(key capabilities for data governance and master data management)**	Connectivity to > 5 data sources	AWS, Microsoft Azure, Google Cloud Platform, and more. Plus, SaaS, packaged apps, and web services
**OpenRefine**<br><br>**(Tool for cleaning and transforming data)**	Connectivity to < 3 data sources	TSV, CSV, *SV, .xls, .xlsx, JSON, XML, RDF as XML and google documents
**DataCleaner (COMMUNITY EDITION - Limited)**<br><br>**(Data profiling, data cleaning, and data integration tool) - offers integration with Pentaho**	Connectivity to > 5 data sources	CSV files, Excel spreadsheets<br>JDBC, MySQL, PostrgreSQL, SQL Server<br>Salesforce, SugarCRM
**DataPreparator**	Connectivity to < 3 data sources	JDBC, XLS

For peer review only

(Preprocessing - data cleaning, transformation, and exploration)		ARFF, DATA, CSV or plain text file format
**Data Match (30-DAY FREE TRIAL)**<br><br>**(visual data cleansing application - a component of Data Ladder)**	Connectivity to > 5 data sources	Access, Apache HBase, Dynamics CRM, Email, Excel, Facebook, JSON, MongoDB, MySQL, Salesforce, SugarCRM, Twitter, XML
**DataMartist (30 DAY FREE TRIAL, STANDARD - $349, PROFESSIONAL - $995)**<br><br>**(Visual, data profiling and data transformation tool)**	Connectivity to > 5 data sources	SQL Server, Oracle, MySQL, ODBC, MS Access, Excel Spreadsheets, Delimited text files including CSV data
**Pentaho Kettle (COMMUNITY EDITION - Limited)**<br><br>**(ETL Tool) Integrates with WEKA (Data Profiling)**	Connectivity to > 5 data sources	Oracle, PostgreSQL, Redshift, SAP, SQLite, SparkSQL, Sybase, Teradata, UniVerse, Verica, Cloudera Impala, Hypersonic, H2 and more
**SQL Power Architect (COMMUNITY EDITION - Limited)**<br><br>**(Data Modeling & Profiling Tool)**	Connectivity to > 5 data sources	JDBC, PostgreSQL, SQL, MySQL, HSSQLDB, Oracle, DB2, HSQLDB, SQLstream, H2, Derby
**SQL Power DqGuru**	Connectivity to > 5 data sources	JDBC, Oracle, Postgress, MySQL, Sybase and more

For peer review only

**(COMMUNITY EDITION - Limited)**<br><br>**(Data Cleansing & MDM Tool)**		
**DQ Analyzer (COMMUNITY EDITION - Limited) (Data profiling tool)**	Connectivity to > 5 data sources	Oracle, MS SQL, DB2, Sybase, Teradata, MySQL, Apache Derby, PostgreSQL<br>CSV, TXT, and XLS(X)
**Pimcore**<br><br>**(Data Management, Integration, PIM, MDM, DAM)**	*Unable to collect during study*	*Unable to collect during study*
**CytoScape**<br><br>**(software platform for visualizing molecular interaction networks and biological pathways)**	*Unable to collect during study*	Simple interaction file (SIF or .sif format), Graph Markup Language (GML or .gml format), XGMML (extensible graph markup and modelling language), SBML, BioPAX, PSI-MI Level 1 and 2.5, Delimited text, Excel Workbook (.xls)
**Anaconda**<br><br>**(data science platform)**	Connectivity to > 5 data sources	Multiple Python Connectors
**Pyxplorer**<br><br>**(a simple tool that allows interactive profiling of datasets)**	Connectivity to < 5 data sources	Hive, Impala, MySQL
**MobyDQ**<br><br>**(Testing tool - aims to automate Data Quality checks**	Connectivity to > 5 data sources	Cloudera Hive, MariaDB, Microsoft SQL Server, MySQL, Oracle, PostgreSQL, SQLite, Teradata, Snowflake, Hortonworks Hive

**during data processing)**		

For peer review only

Supplemental Material 2. A Data profiling report produced by Pandas Profiling (Python).

Overview

Overview Reproduction Warnings 32

Dataset statistics

Number of variables	21
Number of observations	10
Missing cells	5
Missing cells (%)	2.4%
Duplicate rows	0
Duplicate rows (%)	0.0%
Total size in memory	1.8 KiB
Average record size in memory	180.8 B

Variable types

CAT	14
NUM	7

CITY
Categorical

HIGH CORRELATION
MISSING

Distinct count	7
Unique (%)	77.8%
Missing	1
Missing (%)	10.0%
Memory size	80.0 B

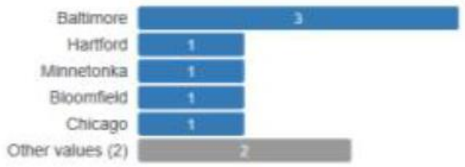

Toggle details

Common Values Length

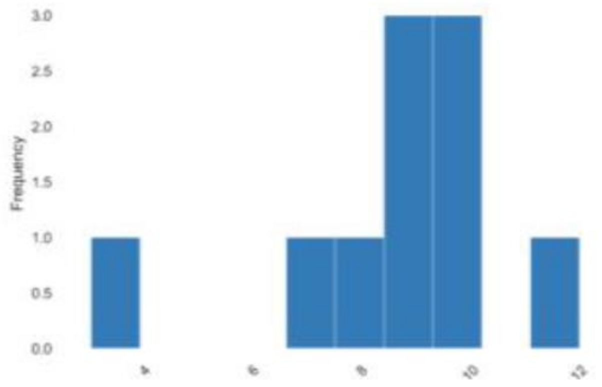

Length

Max length	12
Median length	9
Mean length	8.7
Min length	3

REVENUE
Real number ($\mathbb{R}_{\geq 0}$)

UNIQUE
ZEROS

Distinct count	10	Mean	87811405.0
Unique (%)	100.0%	Minimum	0.0
Missing	0	Maximum	154184100.0
Missing (%)	0.0%	Zeros	1
Infinite	0	Zeros (%)	10.0%
Infinite (%)	0.0%	Memory size	80.0 B

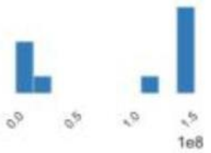

Toggle details

Statistics Histogram(s) Common values Extreme values

Quantile statistics

Minimum	0
5-th percentile	587250
Q1	10433062.5
median	129576100
Q3	142068150
95-th percentile	153313215
Maximum	154184100
Range	154184100
Interquartile range (IQR)	131635087.5

Descriptive statistics

Standard deviation	70229707.73
Coefficient of variation (CV)	0.7997788867
Kurtosis	-2.177497116
Mean	87811405
Median Absolute Deviation (MAD)	23640350
Skewness	-0.4427638806
Sum	878114050
Variance	4.932211848e+15

Correlations

Pearson's r Spearman's ρ Kendall's τ Phik (φk) Cramér's V (φc)

Toggle correlation descriptions

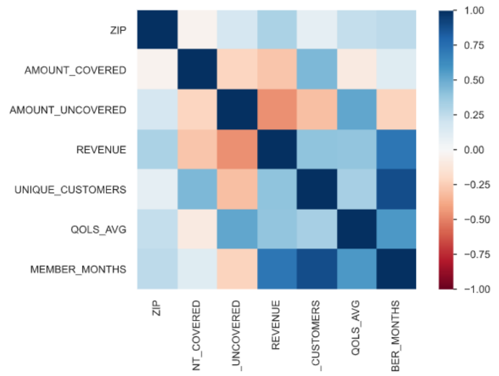

Pearson's r

The Pearson's correlation coefficient (*r*) is a measure of linear correlation between two variables. It's value lies between -1 and +1, -1 indicating total negative linear correlation, 0 indicating no linear correlation and 1 indicating total positive linear correlation. Furthermore, *r* is invariant under separate changes in location and scale of the two variables, implying that for a linear function the angle to the x-axis does not affect *r*.

To calculate *r* for two variables *X* and *Y*, one divides the covariance of *X* and *Y* by the product of their standard deviations.

BMJ Open

Evaluation of Freely Available Data Profiling Tools for Health Data Research Application: a functional evaluation review

Journal:	*BMJ Open*
Manuscript ID	bmjopen-2021-054186.R2
Article Type:	Original research
Date Submitted by the Author:	03-Apr-2022
Complete List of Authors:	Gordon, Ben; Health Data Research UK<br>Fennessy, Clara; Health Data Research UK<br>Varma, Susheel; Health Data Research UK<br>Barrett, Jake; Health Data Research UK<br>McCondochie, Enez; Inspirata Ltd<br>Heritage, Trevor; Inspirata Ltd<br>Duroe, Oenone; Inspirata Ltd<br>Jeffery, Richard; Inspirata Ltd<br>Rajamani, Vishnu; Inspirata Ltd<br>Earlam, Kieran; Cystic Fibrosis Trust<br>Banda, Victor; Imperial College London Neonatal Medicine Research Group, Neonatal Data Analysis Unit<br>Sebire, Neil; Health Data Research UK
**Primary Subject Heading**:	Health informatics
Secondary Subject Heading:	Health services research, Health policy
Keywords:	Information management < BIOTECHNOLOGY & BIOINFORMATICS, Health informatics < BIOTECHNOLOGY & BIOINFORMATICS, Information technology < BIOTECHNOLOGY & BIOINFORMATICS

SCHOLARONE™
Manuscripts

I, the Submitting Author has the right to grant and does grant on behalf of all authors of the Work (as defined in the below author licence), an exclusive licence and/or a non-exclusive licence for contributions from authors who are: i) UK Crown employees; ii) where BMJ has agreed a CC-BY licence shall apply, and/or iii) in accordance with the terms applicable for US Federal Government officers or employees acting as part of their official duties; on a worldwide, perpetual, irrevocable, royalty-free basis to BMJ Publishing Group Ltd ("BMJ") its licensees and where the relevant Journal is co-owned by BMJ to the co-owners of the Journal, to publish the Work in this journal and any other BMJ products and to exploit all rights, as set out in our licence.

The Submitting Author accepts and understands that any supply made under these terms is made by BMJ to the Submitting Author unless you are acting as an employee on behalf of your employer or a postgraduate student of an affiliated institution which is paying any applicable article publishing charge ("APC") for Open Access articles. Where the Submitting Author wishes to make the Work available on an Open Access basis (and intends to pay the relevant APC), the terms of reuse of such Open Access shall be governed by a Creative Commons licence – details of these licences and which Creative Commons licence will apply to this Work are set out in our licence referred to above.

Other than as permitted in any relevant BMJ Author's Self Archiving Policies, I confirm this Work has not been accepted for publication elsewhere, is not being considered for publication elsewhere and does not duplicate material already published. I confirm all authors consent to publication of this Work and authorise the granting of this licence.

For peer review only

Evaluation of Freely Available Data Profiling Tools for Health Data Research Application: a functional evaluation review

BEN GORDON[1], CLARA FENNESSY[1], SUSHEEL VARMA[1], JAKE BARRETT[1], ENEZ MCCONDOCHIE[2], TREVOR HERITAGE[2], OENONE DUROE[2], RICHARD JEFFERY[2], VISHNU RAJAMANI[2], KIERAN EARLAM[3], VICTOR BANDA[4], NEIL J SEBIRE[1]

1. Health Data Research UK, London, UK
2. Inspirata Ltd, Tampa, Florida, USA
3. Cystic Fibrosis Trust, London, UK
4. Neonatal Data Analysis Unit, Imperial College London, London, UK

Correspondence:

PROFESSOR NEIL J SEBIRE

Chief Clinical Data Officer, Health Data Research UK

Wellcome Trust, Gibbs Building, 215 Euston Road, London, NW1 2BE

Email: neil.sebire@hdruk.ac.uk

Word Count: 2859

For peer review only - http://bmjopen.bmj.com/site/about/guidelines.xhtml

ABSTRACT

Objectives: To objectively evaluate freely available data profiling software tools using healthcare data.

Design: Data profiling tools were evaluated for their capabilities using publicly available information and data sheets. From initial assessment, several underwent further detailed evaluation for application on healthcare data using a synthetic dataset of 1000 patients and associated data using a common health data model, and tools scored based on their functionality with this dataset.

Setting: Improving the quality of healthcare data for research use is a priority. Profiling tools can assist by evaluating datasets across a range of quality dimensions. Several freely available software packages with profiling capabilities are available but healthcare organizations often have limited data engineering capability and expertise.

Participants: 28 profiling tools, eight undergoing evaluation on synthetic dataset of 1000 patients.

Results: Of 28 potential profiling tools initially identified, eight showed high potential for applicability with healthcare datasets based on available documentation, of which two performed consistently well for these purposes across multiple tasks including determination of completeness, consistency, uniqueness, validity, accuracy and provision of distribution metrics.

Conclusions: Numerous freely available profiling tools are serviceable for potential use with health datasets, of which at least two demonstrated high performance across a range of technical data quality dimensions based on testing with synthetic health dataset and common data model. The appropriate tool choice depends on factors including underlying organizational infrastructure, level of data engineering and coding expertise, but there are

For peer review only

For peer review only - http://bmjopen.bmj.com/site/about/guidelines.xhtml

freely available tools helping profile health datasets for research use and inform curation activity.

For peer review only

For peer review only - http://bmjopen.bmj.com/site/about/guidelines.xhtml

Strengths and limitations of this study

- We are not aware of any other publication reviewing open and open-source data profiling tools using this level of rigour.
- A range of freely available data profiling tools are capability mapped regarding utility for profiling health data sets.
- Use of such data profiling software tools can help improve data quality by understanding the technical dimensions of a given health data set
- There may be other potentially suitable tools in existence that were not discovered and evaluated.
- It was not always possible to find out information on individual tools from available documentation.

For peer review only

For peer review only - http://bmjopen.bmj.com/site/about/guidelines.xhtml

INTRODUCTION

Health Data Research UK's mission is to unite the UK's health data to enable discoveries that improve people's lives. [1] One aspect of this activity is the ambition to provide a consistent view on the utility of particular datasets for specific purposes through an Innovation Gateway. [2] This would allow users to understand whether a dataset is likely to meet their needs, ahead of requesting access. One important aspect of the utility of a dataset relates to the technical dimensions of data quality, [3] as the consistent use of data quality metrics can facilitate comparison between datasets and, in addition, can demonstrate areas of potential improvement for data custodians. Data quality is frequently cited as a challenge in undertaking health research, as well as for other uses of health data. [4] Commonly used data quality dimensions in health include completeness, consistency, uniqueness, validity, accuracy, and timeliness. [5]

There are a variety of approaches used for establishing the quality of health data, hindering wider use of data due to challenges in understanding and communicating the usefulness of the data. [6]In addition to domain-specific subject matter expertise, semi-automated analysis of datasets using data quality profiling software tools can assist the process, supporting increased awareness of data quality of datasets, completeness and consistency of data submissions, improved reliability, accuracy and auditability and ultimately 'better' more usable data over time. Data profiling is the process of reviewing source data, understanding the structure, content and interrelationships of elements, examining records to discover errors/issues relating to content and format, and understanding data distributions and other factors. [7] It is seen as an important step towards improving the quality and usefulness of data. [8] There are many challenges in profiling data, depending on the structure and format of the underlying data. [9]

Many software tools are available, with varied applicability and data profiling capability for healthcare data. The aims of this study were to identify and evaluate functionality and usability of existing openly available (either open source or free-to-use) data quality assessment tools for potential users across the health data research community with specific focus on data profiling capabilities. There are many studies looking at the effectiveness of

For peer review only - http://bmjopen.bmj.com/site/about/guidelines.xhtml

tools for data analysis, but few that focus on data profiling or curation. [10] This research often focuses on libraries or packages available to users of a specific coding language. [11], [12] Through this research we wanted to provide resources available to understand the data itself.

Technical data quality metrics across the dimensions described above represents only a subset of overall characteristics to describe usefulness, or utility, of a dataset. Other factors, such as source, provenance, time period, geographical coverage, etc may determine the utility for a particular project, independent of any technical data quality metrics. [13] Furthermore, data in a given data set may have an acceptable level of quality for some contexts or use cases, for example a student technical project, but the same data may be inadequate in other contexts, such as use for healthcare regulatory purposes, based on a range of factors. The concept of overall evaluation of dataset utility for specific use cases is becoming more widely recognised. [14]

For peer review only - http://bmjopen.bmj.com/site/about/guidelines.xhtml

METHODS

Study design

In order to evaluate existing freely available data profiling tools for potential use with health datasets, a desk-based activity was performed. This first required the identification of as many tools as possible that would be available without cost, followed by an initial evaluation of the identified tools against a range of broad criteria based on publicly available information regarding the tool functionalities. Following this evaluation, tools which scored highly in the areas of most interest for profiling of health datasets were tested on a synthetic health dataset to evaluate their capability in an objective way.

Identification of tools

An initial scoping exercise was conducted to identify data profiling tools that were freely available. This included tools that were open-source and those that were proprietary but freely available (or having a functional freely available version). The tools were identified through web searches, with search terms of “data processing tools”, “data quality tools”, “data profiling tools” and “data curation tools”and inclusion criteria being the absence license restrictions, cost, lack of expert level user requirements and appropriateness of functionality as relates to health data quality. This was supplemented by discussion with individuals currently working in the sector and involved in data profiling and curation. This process resulted in 28 potential tools for initial evaluation, some of which were generic tools.

Initial Evaluation

In order to evaluate the tools, a general comparison matrix was developed based on criteria used previously for evaluating data quality tools. [15] EM identified individual functions drawing from Gartner and DAMA criteria, as well as suggesting further functions, which could be categorised into functional areas and major categories. EM and TH developed an initial categorisation of functional areas and major categories, and this was refined in collaboration with BG, SV and NJS. The scoring matrix was developed as a feature tree, comprising five major categories and fourteen minor functional areas, and a maximum score allocated for each area. The 28 tools were initially compared and categorized against the matrix using information from the available product documentation and data sheets.(Table 1)

For peer review only - http://bmjopen.bmj.com/site/about/guidelines.xhtml

For peer review only

For peer review only - http://bmjopen.bmj.com/site/about/guidelines.xhtml

Table 1. Detailed Scoring Criteria per Feature

FEATURE TREE			SCORE
**Data Ingestion and Integration**	*Data Consolidation*	Connectivity to N data sources	5
		Data Extraction, Transformation and Loading (ETL) and ETL support	5
		Data modelling	5
	Data Propagation	Data flow orchestration, Enterprise Application Integration (EAI), exchange of messages and transactions	5
		Enterprise Data Replication (EDR), transfer large amounts of data between databases	5
		Versioning and file management	5
	Data Virtualization	Data access	5
	Data Federation	Enterprise Information Integration (EII)	5
		**Total**	**40**
**Data Preparation and Cleaning**	*Parsing and Standardization*	Tagging data with keywords, descriptions or categories	5
		Data scrubbing/cleansing/handling blank values/reformatting values/threshold checking	5
		Data enhancement/enrichment/curation	5
		Natural Language Processing	5
		Address validation/geocoding	5
		Master data management	5
		Data masking	5
	Identity Resolution, Linkage, Merging & Consolidation	Data de-duping	5
		Machine Learning (ML) / training a statistical model	5
		Data aggregation	5
		Data binning	5
		Grouping similar data / clustering	5
		Outlier detection and removal	5
	Master Reference Data Management	"Hub" infrastructure to source and distribute master/reference data	5
		Master data versioning based on data history and timelines	5
		Workflow integrations to steward and publish the master/reference data	5
		Graph data stores to define relationships for creating a flexible knowledge graph	5
		Accessible API for real-time access to shared reference data	5
		**Total**	**90**
**Data Profiling, Exploration/**	*Relationship discovery*	Cross table redundancy analysis	5
		Performing data quality assessment, risk of performing joins on the data	5

For peer review only

For peer review only - http://bmjopen.bmj.com/site/about/guidelines.xhtml

**Pattern Detection**		Identifying distributions, key candidates, foreign-key candidates, functional dependencies, embedded value dependencies, and performing inter-table analysis.	5
	Content discovery	Data pattern discovery	5
		Domain analysis	5
		Discovering metadata and assessing its accuracy	5
	Structure discovery	Column value frequency analysis & statistics, collecting descriptive statistics like min, max, count and sum.	5
		Table structure analysis, collecting data types, length and recurring patterns.	5
		Drill-through analysis	5
		**Total**	**45**
**Data Monitoring**	*Monitoring & Alerting*	Time series data identified and collection by metric name and key/value pairs	5
		Flexible query language to leverage this dimensionality	5
		Graphing and dashboarding support	5
		**Total**	**15**
**Data Use**	*Metadata Management*	Concept identification and naming	5
		Data categorization	5
		Lineage	5
		Relationship with other metadata	5
		Comments and remarks	5
		Data statistics (profiles)	5
		Knowledge graph	5
	Privacy & Security	Data anonymization	5
		Role based access control	5
		Secure environment setup and deployment	5
		Container based deployment	5
	Data Mining	Interactive data visualization	5
		Visual programming and analysis	5
		Visual illustrations & training documentation	5
		Sample data / generate fake data	5
		Add-ons and extension functionality	5
		**Total**	**80**

Each tool was ranked based on key capabilities required to address the profiling aspects of data quality using the feature tree and scoring. Tools were assigned the available weighted scoring based on the ability to provide the function described, according to the information available. Each feature was scored using a binary system, either 0 or 5. An exception to this

For peer review only - http://bmjopen.bmj.com/site/about/guidelines.xhtml

rule is the "Connectivity to N data sources" where this feature is scored 3, 4, and 5 when a tool has connectivity to < 3, < 6, and > 5 data sources, respectively. Scores for each of the five major category areas were converted to a percentage of the total available score for that area.

In-depth evaluation

Following the initial evaluation, eight tools scored were selected for further, in-depth evaluation based on the data profiling major category score and functions (the focus of this process was to evaluate data profiling capabilities; other potential functionalities were recorded for interest as above but not used for ranking). The selected tools included: Knime, DataCleaner, Orange, WEKA, Pandas-profiling (Python), Aggregate Profiler, Talend Open Studio for Data Quality, WhiteRabbit. (Rapid Miner and DQ Analyzer were excluded since they were limited free versions of paid-for tools. Since two python tools, Pandas Profiling and Anaconda, scored highly for profiling, only Pandas profiling was further evaluated since it is explicitly intended for data profiling. Finally, WhiteRabbit, Talend Open Studio for Data Quality and Aggregate Profiler were also evaluated since they were identified as being used by the HDR UK community). To evaluate these tools for their data profiling performance and capability, synthetic data sets were created using the open source tool, Synthea to generate CSV files and SQL Database adhering to the Observational Medical Outcomes Partnership Common Data Model (an internationally adopted data standard) containing 1000 patients and related clinical data and the tools run on this dataset. [16]Synthea allows generation of fully synthetic datasets which broadly conform to the data types and values expected in a 'real' health dataset but with no risk of patient data identification. [17] To evaluate performance and scalability of each tool an additional synthetic dataset of 1.3 million records was also generated.

Each of the shortlisted open-source data profiling tools were evaluated based on how possible it was to execute common specific profiling functions as described in the tool documentation decided based on the Gartner reports. [18]

Further to the initial evaluation, the shortlisted tools were evaluated in-depth based on the ability to deliver data profiles against core DAMA UK data quality dimensions, [3] including

For peer review only - http://bmjopen.bmj.com/site/about/guidelines.xhtml

completeness (the proportion of stored data against the potential of 100% complete), consistency (the absence of difference, when comparing two or more representations of a thing against a definition), uniqueness (nothing recorded more than once based upon how that thing is identified), validity (data are valid if it conforms to the syntax (format, type, range) of its definition), accuracy (the degree to which data correctly describes the object or event being described) and timeliness (the degree to which data represent reality from the required point in time). For each data profiling functionality, tools were run and subjectively scored on a scale of 0-5 according to a semi-structured scale (0=unable to process, 1=most requirements not achieved, 2=some requirements not achieved, 3=meets core requirements, 4=meets and exceeds some requirements, 5=significantly exceeds core requirements).

The suitability of the tools for potential future use by other parties was estimated based on feedback from volunteers from the HDR UK community testing selected tools on their local datasets and providing a qualitative comment on usability. Formal evaluation of the tools of a range of real-world health datasets in a range of environments was outside the scope of this study.

Patient and Public Involvement

Patients and/or the public were not involved in the design, or conduct, or reporting, or dissemination plans of this research.

For peer review only - http://bmjopen.bmj.com/site/about/guidelines.xhtml

RESULTS

Initial evaluation

The initial 28 tools evaluated are shown in Online Supplemental Material 1 along with scores in the various data quality task categories with detailed results for data profiling functionality. The overall results of the initial scoring are shown in Figure 1, where scores have been normalised to a maximum of 1 to support initial inspection.

Subsequent evaluation

Based on the in-depth review of the selected eight tools to evaluate their ability to deliver key functions, the Python library, Pandas Profiling, was identified as possessing the most versatile functionality, able to complete all 30 of the identified profiling functions on the synthetic dataset for testing. The next most versatile tool, Knime, was able to perform 19 such tasks. Across the functionality types, Single Column – Cardinalities was one that the most tools were capable of delivering, with all tools able to deliver three of the functions in this type. The functionality type that was least well served by the tools was Dependencies, with only Pandas Profiling able to deliver any of these functions.(Table 2)

Table 2. Specific Data Profiling Tool Functionalities Evaluated

*** Key:**
K=Knime; DC=DataCleaner; O=Orange; W=WEKA; PP=Pandas Profiling (Python); AP=Aggregate Profiler; TOS=Talend Open Studio for Data Quality; WR=WhiteRabbit

FUNCTIONALITY TYPE	FUNCTION	DATA PROFILING TOOLS CAPABLE OF NATIVELY EXECUTING FUNCTION *							
		K	DC	O	W	PP	AP	TOS	WR
**Single Column – Cardinalities** REFERS TO THE UNIQUENESS OF DATA VALUES CONTAINED IN A PARTICULAR COLUMN (ATTRIBUTE) OF A TABLE (ENTITY)	*Number of rows*	✓	✓	✓	✓	✓	✓	✓	✓
	Number of nulls	✓	✓	✓	✓	✓	✓	✓	✓
	Percentage of nulls	✓		✓	✓	✓		✓	✓
	Number of distinct values (cardinality)	✓	✓	✓	✓	✓	✓	✓	✓
	Percentage of distinct values (Number of distinct values divided by the number of rows)	✓			✓	✓		✓	
**Single Column - Value distributions** PRESENTS AN ORDERING OF THE RELATIVE FREQUENCY (COUNT	*Frequency histograms (equi-width, equi-depth, etc.)*	✓				✓			
	Minimum and maximum values in a numeric column	✓	✓	✓		✓	✓	✓	✓

For peer review only - http://bmjopen.bmj.com/site/about/guidelines.xhtml

AND PERCENTAGE) OF THE ASSIGNMENT OF DISTINCT VALUES	*Constancy (Frequency of most frequent value divided by number of rows)*	✓				✓		✓	
	Quartiles (3 points that divide the numeric values into 4 equal groups)	✓	✓			✓	✓	✓	✓
	Distribution of first digit in numeric values (to check Benford's law)	✓				✓		✓	
**Single Column - Patterns, datatypes, and domains** REFERS TO THE DISCOVERY OF PATTERNS AND DATA TYPES	*Basic types (e.g., numeric, alphanumeric, date, time)*	✓				✓			
	DBMS-specific data type (e.g., varchar, timestamp)	✓	✓			✓	✓	✓	✓
	Measurement of Value length (minimum, maximum, average, median)	✓	✓	✓		✓	✓		✓
	Maximum number of digits in numeric values	✓	✓			✓	✓		
	Maximum number of decimals in numeric values	✓				✓	✓		
	Histogram of value patterns (Aa9...)	✓	✓			✓		✓	
	Generic semantic data type (e.g., code, date/time, quantity, identifier)	✓	✓			✓		✓	
	Semantic domain (e.g., credit card, first name, city)	✓	✓			✓		✓	
**Dependencies** DETERMINES THE DEPENDENT RELATIONSHIPS WITHIN A DATA SET	*Unique column combinations (UCCs) (key discovery)*					✓			
	Relaxed unique column combinations					✓			
	Inclusion dependencies (INDs) (foreign key discovery)					✓			
	Relaxed inclusion dependencies					✓			
	Functional dependencies					✓			
	Conditional functional dependencies					✓			
**Advanced Multi Column profiling** DETERMINES THE SIMILARITIES AND DIFFERENCES IN SYNTAX AND DATA TYPES BETWEEN TABLES (ENTITIES) TO DETERMINE WHICH DATA MIGHT BE REDUNDANT AND WHICH COULD BE MAPPED TOGETHER	*Correlation analysis*			✓		✓	✓		
	Association rule mining					✓			
	Cluster analysis					✓			
	Outlier detection	✓		✓		✓			
	Exact duplicate tuple detection		✓			✓		✓	
	Relaxed duplicate tuple detection		✓			✓		✓	
	**Total**	**19**	**13**	**8**	**5**	**30**	**10**	**15**	**8**

The tools were further evaluated based on their ability to deliver data profiles against the DAMA dimensions.(Figure 2) Pandas Profiling achieved significantly greater results compared

For peer review only - http://bmjopen.bmj.com/site/about/guidelines.xhtml

to the other tools, scoring 110 of the available points, compared to the next highest tool, Knime, with 61 points. Of the tools examined, WhiteRabbit had the least comprehensive functionality in this area, able only to provide information against the Completeness element. Across the different elements, Completeness was best served by the profiling tools, with all tools able to provide some functionality in this area. The least well-served element was Consistency, with only Pandas Profiling able to provide any output for this element. Online Supplemental Material 2 shows the profile reporting information produced by Pandas Profiling with features including basic dataset statistics overview, reports on specific numerical or categorical variables, and correlations between variables.

Links for all tools tested are available here (https://github.com/HDRUK/data-utility-tools).

User testing feedback

To provide anecdotal feedback on the usability of the tools, five of the eight tools (DataCleaner, Orange, MobyDQ, Knime and Aggregate profiler) were tested by volunteers from the Cystic Fibrosis Trust and the Neonatal Medicine Research Group. These tools were selected for testing based of the volunteer's ability and the resources available to run them.

MobyDQ and Aggregate Profiler both presented difficulties to the volunteers due to challenges installing and running the software. MobyDQ failed to authenticate due to issues with private keys and Aggregate Profiler crashed upon attempts to update.

Knime, DataCleaner and Orange could be run successfully by the volunteers. Orange required the local migration of data and installation of two additional modules, and was supported more effectively on Mac OS and Linux than Windows. Knime was fairly resource intensive and initially difficult to use, but was seen to be capable of a range of functions. DataCleaner was reported to be relatively easy to set up and run, even on a Windows machine, and capable of linking to existing databases.

For peer review only

For peer review only - http://bmjopen.bmj.com/site/about/guidelines.xhtml

DISCUSSION

The findings of the present study have demonstrated that numerous openly available data profiling tools are available, with several able to perform well using health datasets. The precise choice of tool for organisations will depend on the data type, model and format, in addition to IT environment, such as Windows or Linux, and expertise with such tools and coding languages, such as Python. Regardless of the tools used, appropriate deployment and dataset evaluation through data profiling should lead to early detection of data quality issues for particular data sets and sources and consequent ability to remediate such issues. The identification of Pandas Profiling as a versatile approach to data profiling is reinforced by the fact that, as a Python library, it can be combined with other tools, such as Orange or Knime, to provide an even more in-depth output.

This study provides a useful resource for individuals anywhere in the world to understand the functionality of freely available data profiling tools for use with health datasets, and put these to use. The creation of an open and persistent resource is a strength of the study. All the outputs of the testing, as well as the generated dataset, are available (https://github.com/HDRUK/data-utility-tools). None of the tested tools are specific to health data, and therefore could be used in any other domain. However, the open nature of the search for the tools, the absence of an indexed repository of these tools was likely non-exhaustive. There may be additional tools that would also have been suitable for this exercise that were not identified during the project. Furthermore, the tools were tested on a synthetic dataset, which was useful for testing functionality, but does not necessarily represent the condition of "real" health data, which may include numerous additional or unexpected errors and anomalies. Ideally, the team would have been able to test the tools on real patient data, but information governance approvals were not possible in the available time and a fully standardised dataset was required to ensure objectivity when comparing tools, hence a controlled synthetic dataset was most appropriate for the present purposes. While some of the tools were tested on real datasets by volunteers (Cystic Fibrosis Trust and Neonatal Data Analysis Unit), this was designed to review the initial views regarding usability of the tool, rather than provide a comparison of the outputs.

For peer review only - http://bmjopen.bmj.com/site/about/guidelines.xhtml

Determining data quality is a complex process and far harder than commonly assumed, especially for high dimensional and longitudinal data such as health data. Data profiling provides the user with an understanding of the inherent technical data quality according to various dimensions within a given dataset but does not, in itself, improve quality. Rather, based on the outcome of data profiling, it will likely be required to utilize one or more data quality tools to remediate issues detected, this being best accomplished by data analysts and/or scientists with subject matter expertise, working close to the original source of the data. While the ability of the tools to be used by individuals with limited experience was not the focus of this research, this would be interesting to explore in future work, particularly because the tool with the broadest capability, Pandas Profiling, was not tested by volunteers. There are a large number of libraries and packages available for coding languages such as Python and R, for example skimr. [19] These resources provide powerful capabilities for analysts, but often require some amount of technical capability, reducing their accessibility to many users.

Further research would be useful to understand the capability of the tools in handling increasingly large sets of data. While the tools were tested against a dataset of over one million patient records, processing time was not compared quantitatively. Further, in a healthcare or health research setting, it is not unusual for a dataset to be several orders of magnitude larger than this. For a tool to be useful in these settings, it should be able to process large datasets, and within a reasonable time.

As referenced in the Introduction, there is a need for greater consistency in how dimensions of data quality are assessed and communicated. The wider adoption of data profiling tools would encourage greater literacy and higher expectations among users of health data. Transparency of current dataset profiles, for example on the Innovation Gateway, would provide an incentive for focused improvement of data, as well as informed decision-making by users. Further work could be done in the presentation of the outputs of data profiling exercises, in order to ascertain the approach that is most conducive to effective data curation.

For peer review only - http://bmjopen.bmj.com/site/about/guidelines.xhtml

Evaluation of a wide range of freely available software tools for data engineering with a focus on data profiling for health care data tested using synthetic datasets has determined that several tools perform highly in a range of tasks appropriate to this use case. By the more widespread use of routine health dataset profiling, and associated remediation, along with other measures to understand and improve dataset utility, we anticipate that the overall quality of health data for research use can be increased.

FUNDING STATEMENT

This work was supported by Medical Research Council capital funding (August 2019). There is no grant number associated with capital fund awards.

CONTRIBUTORSHIP STATEMENT

BG, SV and NS conceived the study. EM, TH, OD, RJ and VR developed the methodology further, evaluated the tools and provided the initial results. KE and VB tested the tools on their own datasets and provided feedback on results. NS, BG, CF and JB prepared and drafted the manuscript. The guarantor of the content is NS.

COMPETING INTERESTS

None declared. EM, TH, OD, RJ, VR were employed by Inspirata Ltd at the time of the work but were contracted by HDR UK to carry out this work independently on behalf of HDR UK.

ETHICS APPROVAL

As a desk-based project, involving no patients or other human subjects, having no relation to clinical protocols and not intending to provide generalisable results, no ethical approval was required.

DATA AVAILABILITY STATEMENT

Data are available upon reasonable request.

FIGURE CAPTION

For peer review only - http://bmjopen.bmj.com/site/about/guidelines.xhtml

Figure 1: Main results of documentation based functionality for data quality categories by tool

Figure 2: Results of profiling tasks using synthetic datasets. KNIME and Pandas performed best for overall data profiling tasks for this healthcare dataset

For peer review only

For peer review only - http://bmjopen.bmj.com/site/about/guidelines.xhtml

References

[1] Health Data Research UK, "Home," HDR UK, [Online]. Available: https://www.hdruk.ac.uk. [Accessed 14 August 2020].

[2] Health Data Research UK, "HDR UK Innovation Gateway," HDR UK, [Online]. Available: https://www.healthdatagateway.org/. [Accessed 12 October 2020].

[3] A. Black and P. v. Nederpelt, "Code for Information Quality 2019," 5 September 2020. [Online]. Available: http://www.dama-nl.org/wp-content/uploads/2020/09/DDQ-Dimensions-of-Data-Quality-Research-Paper-version-1.2-d.d.-3-Sept-2020.pdf. [Accessed 3 February 2022].

[4] T. Botsis, G. Hartvigsen, F. Chei and C. Weng, "Secondary Use of EHR: Data Quality Issues and Informatics Opportunities," *Summit on Translat Bioinforma,* pp. 1-5, 2010.

[5] H. Chen, D. Hailey, N. Wang and P. Yu, "A Review of Data Quality Assessment Methods for Public Health Information Systems," *Int. J. Environ. Res. Public Health,* vol. 11, no. 5, pp. 5170-5270. doi: 10.3390/ijerph110505170, 2014.

[6] M. Mashoufi, H. Ayatollahi and D. Khorasani-Zavareh, "A Review of Data Quality Assessment in Emergency Medical Services," *Open Med Inform J.,* vol. 12, pp. 19-32. doi: 10.2174/1874431101812010019, 2018.

[7] F. Naumann, "Data profiling revisited," *ACM SIGMOD Record,* vol. 42, no. 4, pp. 40-49, 2013.

[8] R. Mahanti, "Critical Success Factors for Implementing Data Profiling," *Software Quality Professional,* vol. 16, no. 2, pp. 13-26, 2014.

[9] Z. Abedjan, L. Golab and F. Naumann, "Profiling relational data: a survey," *The VLDB Journal volume,* vol. 24, pp. 557-581, 2015.

[10] C. A. Barry, "Choosing Qualitative Data Analysis Software: Atlas/ti and Nudist Compared," *Sociological Research Online,* vol. 3, no. 3, pp. 16-28, 1998.

[11] I. Stančin and A. Jović, "An overview and comparison of free Python libraries for data mining and big data analysis," in *42nd International Convention on Information and Communication Technology, Electronics and Microelectronics (MIPRO)*, Opatija, 2019.

[12] M. Staniak and P. Biecek, "The landscape of R packages for automated exploratory data analysis," *arXiv preprint arXiv:1904.02101.*

[13] B. Gordon, J. Barrett, C. Fennessy, C. Cake, A. Milward, C. Irwin, M. Jones and N. Sebire, "Development of a data utility framework to support effective health data curation," *BMJ Health & Care Informatics,* vol. 28, pp. e100303. doi: 10.1136/bmjhci-2020-100303, 2021.

[14] EUnetHTA, "REQueST Tool and its Vision Paper," EUnetHTA, [Online]. Available: https://www.eunethta.eu/request-tool-and-its-vision-paper/. [Accessed 22 October 2020].

For peer review only - http://bmjopen.bmj.com/site/about/guidelines.xhtml

[15] Gartner, "Magic Quandrant Research Methodology," Gartner, 2019. [Online]. Available: https://www.gartner.com/en/research/methodologies/magic-quadrants-research. [Accessed 12 October 2022].

[16] OHDSI (Chapter lead: Clair Blacketer), "Chapter 4 The Common Data Model | The Book of OHDSI," 11 1 2021. [Online]. Available: https://ohdsi.github.io/TheBookOfOhdsi/CommonDataModel.html. [Accessed 3 February 2022].

[17] Synthea, "GitHub - synthetichealth/synthea," GitHub, 31 January 2022. [Online]. Available: https://github.com/synthetichealth/synthea. [Accessed 3 February 2022].

[18] Gartner, "Critical Capabilities for Data Quality Tools," Gartner, 14 May 2019. [Online]. Available: https://www.gartner.com/en/documents/3913549. [Accessed 21 February 2021].

[19] Comprehensive R Archive Network, "Using Skimr," [Online]. Available: https://cran.r-project.org/web/packages/skimr/vignettes/skimr.html. [Accessed 3 April 2021].

For peer review only

For peer review only - http://bmjopen.bmj.com/site/about/guidelines.xhtml

Add Tool Delete Tool	Data Ingestion and Integration	Data Ingestion and Integration	Data Ingestion and Integration	Data Ingestion and Integration	Data Preparation and Cleaning	Data Preparation and Cleaning	Data Preparation and Cleaning	Data Preparation and Cleaning	Data Preparation and Cleaning	Data Profiling, Exploration / Pattern Detection	Data Monitoring	Data Use	Data Use	Data Use	Data Use
	Connectivity	Parsing	Issue resolution and workflow	Architecture and integration	Master Reference Data Management	Standardization and cleansing	Matching, linking and merging	Address validation / geocoding	Data curation and enrichment	Data profiling, measurement and visualization	Monitoring	Metadata management	Usability	DevOps environment	Deployment environment
Knime	0.29	1.00	1.00	0.75	0.00	1.00	1.00	1.00	1.00	1.00	1.00	0.43	0.67	0.00	0.00
Pandas Profiling	0.29	0.00	0.00	0.00	0.00	0.00	0.00	0.00	0.00	1.00	0.00	0.00	0.33	0.00	0.00
Orange	0.29	1.00	1.00	0.25	0.00	0.50	1.00	1.00	0.67	1.00	1.00	0.00	0.67	0.00	0.00
RapidMiner	0.29	1.00	0.50	0.50	0.00	0.50	1.00	0.00	0.33	1.00	1.00	0.00	0.67	0.00	0.00
WEKA	0.18	0.00	0.00	0.00	0.00	0.25	0.80	0.00	0.67	1.00	0.00	0.43	0.17	0.00	0.00
Anonimatron	0.29	0.00	0.00	0.00	0.00	0.00	0.00	0.00	0.00	0.00	0.00	0.00	0.17	0.00	0.00
ARX Data Anonymization	0.29	0.00	0.00	0.00	0.00	0.25	0.00	0.00	0.33	0.00	0.00	0.00	0.33	0.00	0.00
WhiteRabbit	0.59	0.00	0.50	0.25	0.00	0.00	0.00	0.00	0.00	0.11	0.33	0.00	0.33	0.00	0.00
Aggregate Profiler (AP)	0.29	0.00	0.00	0.00	0.00	0.00	0.60	1.00	0.67	0.78	1.00	0.43	0.17	0.00	0.00
Talend Open Studio for Data Integration	0.29	1.00	0.50	0.25	0.00	0.00	0.00	0.00	0.00	0.00	0.00	0.00	0.00	0.00	0.00
Talend Open Studio For Big Data	0.29	1.00	0.50	0.25	0.00	0.00	0.00	0.00	0.00	0.00	0.00	0.00	0.00	0.00	0.00
Talend Open Studio For Data Quality	0.29	1.00	0.00	0.00	0.00	0.25	0.40	0.00	0.67	0.56	0.00	0.00	0.00	0.00	0.00
Talend Open Studio For ESB	0.29	0.00	1.00	0.00	0.00	0.00	0.00	0.00	0.00	0.00	0.00	0.00	0.00	0.00	1.00
Talend Open Studio For MDM	0.29	0.00	0.00	0.00	0.40	0.25	0.00	0.00	0.33	0.00	0.00	0.00	0.17	0.00	0.00
OpenRefine	0.18	1.00	0.00	0.25	0.00	0.25	0.80	0.00	0.00	0.00	0.00	0.00	0.00	0.00	0.00
DataCleaner	0.29	1.00	1.00	0.50	0.00	1.00	1.00	1.00	1.00	1.00	1.00	0.00	0.33	0.00	0.00
DataPreparator	0.18	0.00	0.00	0.25	0.00	0.25	0.60	0.00	0.00	0.00	0.00	0.00	0.00	0.00	0.00
Data Match	0.29	0.00	0.00	0.00	0.00	0.00	0.40	0.00	0.00	0.00	0.00	0.00	0.00	0.00	0.00
DataMartist	0.29	1.00	0.00	0.00	0.00	0.25	0.20	0.00	0.00	0.11	0.00	0.00	0.17	0.00	0.00
Pentaho Kettle	0.29	1.00	0.00	0.00	0.00	0.00	0.00	0.00	0.00	0.00	0.00	0.00	0.00	0.00	0.00
SQL Power Architect	0.29	0.00	0.00	0.25	0.00	0.00	0.00	0.00	0.00	0.00	0.00	0.14	0.00	0.00	0.00
SQL Power DQguru	0.29	0.00	0.00	0.00	0.00	0.50	0.60	1.00	0.00	0.00	0.00	0.00	0.00	0.00	0.00
DQ Analyzer	0.29	0.00	0.00	0.00	0.00	0.00	0.00	0.00	0.00	1.00	0.00	0.00	0.00	0.00	0.00
Pimcore	0.00	0.00	0.00	0.00	1.00	0.25	0.00	0.00	0.00	0.00	0.00	0.00	0.00	0.00	0.00
CytoScape	0.00	0.00	0.00	0.25	0.00	0.00	0.00	0.00	0.00	0.00	0.00	0.14	0.50	0.00	0.00
Anaconda	0.29	0.00	0.00	0.25	0.00	0.00	0.00	0.00	0.33	1.00	1.00	0.00	0.50	0.00	0.00
pyxplorer	0.24	0.00	0.00	0.00	0.00	0.00	0.00	0.00	0.00	0.11	0.00	0.00	0.00	0.00	0.00
MobyDQ	0.29	0.00	0.50	0.00	0.00	0.00	0.00	0.00	0.00	0.11	0.67	0.00	0.00	0.00	0.00

Main results of documentation based functionality for data quality categories by tool

581x311mm (57 x 57 DPI)

Figure 2. Results of profiling tasks using synthetic datasets. KNIME and Pandas performed best for overall data profiling tasks for this healthcare dataset

**0 = Unable to process**<br>**1 = Poor: most or all defined requirements not achieved**<br>**2 = Fair: some requirements not achieved**	**3 = Good: meets requirements**<br>**4 = Excellent: meets or exceeds some requirements**<br>**5 = Outstanding: significantly exceeds core requirements**

Measure (key elements)	White Rabbit	Orange	Knime	WEKA	Aggregate Profiler	Data Cleaner	Pandas (Python)	Talend Open Studio - Data Quality
**COMPLETENESS - The proportion of stored data against the potential of "100% complete"**								
Percentage of requisite information available	2	4	4	3	2	3	5	1
Percent of missing data values (null / empty string)	2	4	4	4	3	3	5	1
Row counts	4	5	4	4	4	3	5	2
Highest and lowest value of key elements	0	3	5	0	0	3	5	1
Number of data values in an unusable state	0	2	2	0	0	3	5	0
**UNIQUENESS - No thing will be recorded more than once based upon how that thing is identified.**								
(Number of things in the real world) - Number of incorrect spellings etc. of same data in an element e.g. address (duplicate values)	0	2	2	0	1	2	5	2
(Number of recodes describing different things) Number of data items in adherence to expected/described data element value (distinct values at ID level)	0	1	2	0	1	2	5	1
(Number of things in real world i.e. duplicates)/(Number of records describing different things i.e. distinct records)	0	3	4	4	1	2	5	1
**TIMELINESS - The degree to which data represent reality from the required point in time.**								
Difference between Lowest date value and Highest Date Value	0	2	4	0	1	2	3	1
Number of records per month	0	1	3	0	0	2	3	0
**VALIDITY - Data are valid if it conforms to the syntax (format, type, range) of its definition.**								
Percentage of data values that comply with the specified formats (data types, ranges etc.)	0	1	3	0	0	4	5	2
Percentage of data values that don't comply to specified formats	0	0	1	0	0	1	4	0
Number of Missing values indicated e.g. with fill values	0	4	4	0	4	3	5	2
Number of Values in Specified Range	0	0	3	0	0	3	4	0
Number of values not in Specified Range	0	0	2	0	0	3	3	0
**ACCURACY - The degree to which data correctly describes the "real world" object or event being described.**								
Number of accurate data values	0	3	3	0	2	0	5	2
Number of inaccurate data values	0	0	0	0	0	0	5	0
Actual data value count versus predicted data value count	0	0	0	0	0	0	3	0
Number of rows and columns against expectations	0	0	0	0	0	0	3	0
Number of duplicates at ID level	0	4	4	4	3	3	5	3
Number of blank columns, large % of blank data, high % of same data	0	3	4	0	2	0	5	2
Distribution across various segments	0	3	0	0	0	0	5	0
Outliers on key variables	0	3	2	0	0	0	4	0
((Count of accurate objects)/ (Count of accurate objects + Counts of inaccurate objects)	0	1	1	0	0	0	3	0
**CONSISTENCY - The absence of difference, when comparing two or more representations of a thing against a definition.**								
Analysis of pattern and/or value frequency	0	0	0	0	0	0	5	0
**TOTAL SCORES**	8	49	61	19	24	42	110	21

Supplemental Material 1. List of specific tools evaluated

Tool	Connectivity	Data Sources / File Formats
**Knime** **(Data analytics, profiling, reporting and integration platform)**	Connectivity to > 5 data sources	Simple text formats (CSV, PDF, XLS, JSON, XML, etc.)
		Unstructured data types (images, documents, networks, molecules, etc.)
		Time series data
		Connect to a host of databases and data warehouses to integrate data from Oracle, Microsoft SQL, Apache Hive, and more
		Load Avro, Parquet, or ORC files from HDFS, S3, or Azure
		Access and retrieve data from sources such as Twitter, AWS S3, Google Sheets, and Azure and extended via pandas
**Pandas Profiling (using Pandas I/O)** **(Python module for exploratory data analysis (EDA))**	Connectivity to > 5 data sources	Text: - CSV, fixed-width test files, JSON, HTML, Clipboard, Excel
		Binary: OpenDocument, HDF5 Format, Feather Format, Parqeuet Format, ORC Format, Msgpak, Stata, SAS, SPSS, Python Pickle Format
		SQL, Google BigQuery
**Orange** **(Data visualization, machine learning, data profiling and mining toolkit)**	Connectivity to > 5 data sources	Excel (.xlsx), simple tab-delimited (.txt), comma-separated files (.csv) or Google Sheets document
		distance matrix: Distance File
		predictive model: Load Model
		network: Network File from Network add-on
		images: Import Images from Image Analytics add-on
		several spectroscopy files: Multifile from Spectroscopy add-on
		PostgreSQL, SQL, online repository, and extended via pandas
**RapidMiner (LIMITED FREE VERSION)** **(Integrated environment for data preparation, machine learning, deep learning, text mining, and predictive analytics)**	Connectivity to > 5 data sources	Files: CSV, Stata, Hyper (Tableau), XLS, XML, QLikView, and more
		SQL: AccessDB, HSQLDB, Microsoft SQL Server (JTDS / Microsoft), MySQL, Oracle, PostgreSQL, Sybase
		NoSQL: Cassandra, MongoDB, Solr, Splunk (read only)
		Cloud services: Amazon S3, Azure blog and data lake, Dropbox, Google, Salesforce, Twitter, Zapier, Salesforce
**WEKA** **(Machine learning**	Connectivity to < 3 data sources	Arff, JSON, CSV, xrff, dat, data, names, and more
		Database using ODBC

For peer review only

**software to solve data mining problems)**		
**Anonimatron**<br><br>**(Pseudonymizes datasets)**	Connectivity to > 5 data sources	Oracle, PostgreSQL, MySQL, DB2, MsSQL, Cloudscape, Pointbase, Firebird, IDS, Informix, Enhydra, Interbase, Hypersonic, jTurbo, SQLServer and Sybase
**ARX Data Anonymization**<br><br>**(Scalable Data Anonymization Tool - supports multiple privacy models)**	Connectivity to > 5 data sources	CSV files, MS Excel spreadsheets<br>Relational database systems, such as MS SQL, DB2, MySQL or PostgreSQL
**WhiteRabbit**<br><br>**(Tool to help prepare for ETLs of healthcare datasets)**	Connectivity to > 5 data sources	comma-separated text files<br>MySQL, SQL Server, Oracle, PostgreSQL, Microsoft APS, Microsoft Access, Amazon RedShift, Google BigQuery
**Aggregate Profiler (AP)**<br><br>**(Data profiling and analysis tool)**	Connectivity to > 5 data sources	XML, XLS or CSV format, PDF export<br>Teiid, Mysql, Oracle, Postgres, Access, Db2, SQL Server certified Big data support - HIVE
**Talend Open Studio for Data Integration (LIMITED FREE VERSION)**<br><br>**(Data integration and ETL)**	Connectivity to > 5 data sources	More than 900 pre-built connectors and components for Oracle, Teradata, Microsoft SQL server, Marketo, Salesforce, NetSuite, SAP, Microsoft Dynamics, Sugar CRM, Dropbox, Box, SMTP, FTP/SFTP, LDAP, and more
**Talend Open Studio for Big Data (LIMITED FREE VERSION)**<br><br>**(ETL for large and diverse data sets)**	Connectivity to > 5 data sources	Cloud: Amazon Web Services (AWS), Microsoft Azure, Google Cloud Platform, and more<br>RDBMS: Oracle, Teradata, Microsoft SQL server, and more<br>SaaS: Marketo, Salesforce, NetSuite, and more<br>Packaged Apps: SAP, Microsoft Dynamics, Sugar CRM, and more<br>Technologies: Dropbox, Box, SMTP, FTP/SFTP, LDAP, and more

For peer review only

**Talend Open Studio for Data Quality (LIMITED FREE VERSION)**<br><br>**(Assesses accuracy and integrity of data - Data Profiling Tool)**	Connectivity to > 5 data sources	Local or remote file that can be imported into the Talend Data Preparation tool (or from a database connection or other data sources, although not in the context of the Free Desktop version).<br>Excel or CSV file<br>90+ data sources and scale with Stitch Data Loader - https://www.talend.com/products/pricing-model/
**Talend Open Studio for ESB (LIMITED FREE VERSION)**	Connectivity to > 5 data sources	Cloud: Amazon Web Services (AWS), Microsoft Azure, Google Cloud Platform, and more<br>RDBMS: Oracle, Teradata, Microsoft SQL server, and more<br>SaaS: Marketo, Salesforce, NetSuite, and more<br>Packaged Apps: SAP, Microsoft Dynamics, Sugar CRM, and more<br>Technologies: Dropbox, Box, SMTP, FTP/SFTP, LDAP, and more
**Talend Open Studio for MDM (LIMITED FREE VERSION)**<br><br>**(key capabilities for data governance and master data management)**	Connectivity to > 5 data sources	AWS, Microsoft Azure, Google Cloud Platform, and more. Plus, SaaS, packaged apps, and web services
**OpenRefine**<br><br>**(Tool for cleaning and transforming data)**	Connectivity to < 3 data sources	TSV, CSV, *SV, .xls, .xlsx, JSON, XML, RDF as XML and google documents
**DataCleaner (COMMUNITY EDITION - Limited)**<br><br>**(Data profiling, data cleaning, and data integration tool) - offers integration with Pentaho**	Connectivity to > 5 data sources	CSV files, Excel spreadsheets<br>JDBC, MySQL, PostrgreSQL, SQL Server<br>Salesforce, SugarCRM
**DataPreparator**	Connectivity to < 3 data sources	JDBC, XLS

For peer review only

For peer review only - http://bmjopen.bmj.com/site/about/guidelines.xhtml

**(Preprocessing - data cleaning, transformation, and exploration)**		ARFF, DATA, CSV or plain text file format
**Data Match (30-DAY FREE TRIAL)**<br><br>**(visual data cleansing application - a component of Data Ladder)**	Connectivity to > 5 data sources	Access, Apache HBase, Dynamics CRM, Email, Excel, Facebook, JSON, MongoDB, MySQL, Salesforce, SugarCRM, Twitter, XML
**DataMartist (30 DAY FREE TRIAL, STANDARD - $349, PROFESSIONAL - $995)**<br><br>**(Visual, data profiling and data transformation tool)**	Connectivity to > 5 data sources	SQL Server, Oracle, MySQL, ODBC, MS Access, Excel Spreadsheets, Delimited text files including CSV data
**Pentaho Kettle (COMMUNITY EDITION - Limited)**<br><br>**(ETL Tool) Integrates with WEKA (Data Profiling)**	Connectivity to > 5 data sources	Oracle, PostgreSQL, Redshift, SAP, SQLite, SparkSQL, Sybase, Teradata, UniVerse, Verica, Cloudera Impala, Hypersonic, H2 and more
**SQL Power Architect (COMMUNITY EDITION - Limited)**<br><br>**(Data Modeling & Profiling Tool)**	Connectivity to > 5 data sources	JDBC, PostgreSQL, SQL, MySQL, HSSQLDB, Oracle, DB2, HSQLDB, SQLstream, H2, Derby
**SQL Power DqGuru**	Connectivity to > 5 data sources	JDBC, Oracle, Postgress, MySQL, Sybase and more

For peer review only

**(COMMUNITY EDITION - Limited)**<br><br>**(Data Cleansing & MDM Tool)**		
**DQ Analyzer (COMMUNITY EDITION - Limited) (Data profiling tool)**	Connectivity to > 5 data sources	Oracle, MS SQL, DB2, Sybase, Teradata, MySQL, Apache Derby, PostgreSQL<br>CSV, TXT, and XLS(X)
**Pimcore**<br><br>**(Data Management, Integration, PIM, MDM, DAM)**	*Unable to collect during study*	*Unable to collect during study*
**CytoScape**<br><br>**(software platform for visualizing molecular interaction networks and biological pathways)**	*Unable to collect during study*	Simple interaction file (SIF or .sif format), Graph Markup Language (GML or .gml format), XGMML (extensible graph markup and modelling language), SBML, BioPAX, PSI-MI Level 1 and 2.5, Delimited text, Excel Workbook (.xls)
**Anaconda**<br><br>**(data science platform)**	Connectivity to > 5 data sources	Multiple Python Connectors
**Pyxplorer**<br><br>**(a simple tool that allows interactive profiling of datasets)**	Connectivity to < 5 data sources	Hive, Impala, MySQL
**MobyDQ**<br><br>**(Testing tool - aims to automate Data Quality checks**	Connectivity to > 5 data sources	Cloudera Hive, MariaDB, Microsoft SQL Server, MySQL, Oracle, PostgreSQL, SQLite, Teradata, Snowflake, Hortonworks Hive

For peer review only

**during data processing)**		

For peer review only

Supplemental Material 2. A Data profiling report produced by Pandas Profiling (Python).

Overview

Overview | Reproduction | Warnings 32

Dataset statistics

Number of variables	21
Number of observations	10
Missing cells	5
Missing cells (%)	2.4%
Duplicate rows	0
Duplicate rows (%)	0.0%
Total size in memory	1.8 KiB
Average record size in memory	180.8 B

Variable types

CAT	14
NUM	7

CITY
Categorical

HIGH CORRELATION
MISSING

Distinct count	7
Unique (%)	77.8%
Missing	1
Missing (%)	10.0%
Memory size	80.0 B

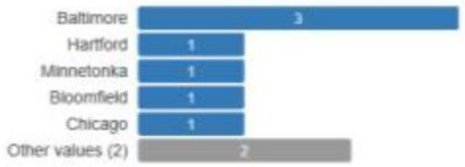

Toggle details

Common Values | Length

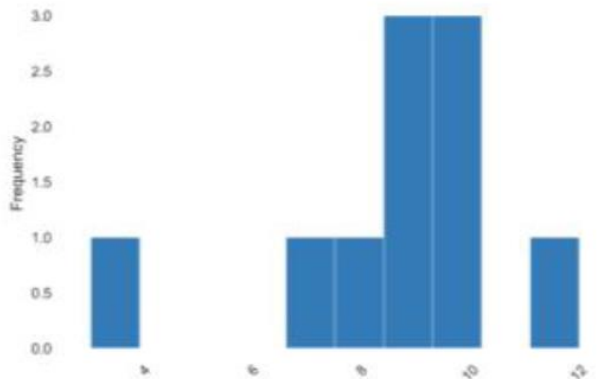

Length

Max length	12
Median length	9
Mean length	8.7
Min length	3

REVENUE

Real number ($\mathbb{R}_{\geq 0}$)

UNIQUE
ZEROS

Distinct count	10
Unique (%)	100.0%
Missing	0
Missing (%)	0.0%
Infinite	0
Infinite (%)	0.0%

Mean	87811405.0
Minimum	0.0
Maximum	154184100.0
Zeros	1
Zeros (%)	10.0%
Memory size	80.0 B

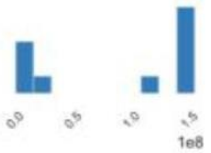

Toggle details

Statistics | Histogram(s) | Common values | Extreme values

Quantile statistics

Minimum	0
5-th percentile	587250
Q1	10433062.5
median	129576100
Q3	142068150
95-th percentile	153313215
Maximum	154184100
Range	154184100
Interquartile range (IQR)	131635087.5

Descriptive statistics

Standard deviation	70229707.73
Coefficient of variation (CV)	0.7997788867
Kurtosis	-2.177497116
Mean	87811405
Median Absolute Deviation (MAD)	23640350
Skewness	-0.4427638806
Sum	878114050
Variance	4.932211848e+15

Correlations

Pearson's r | Spearman's ρ | Kendall's τ | Phik (φk) | Cramér's V (φc)

Toggle correlation descriptions

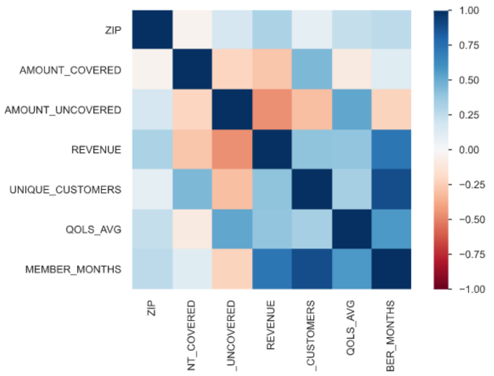

Pearson's r

The Pearson's correlation coefficient (*r*) is a measure of linear correlation between two variables. It's value lies between -1 and +1, -1 indicating total negative linear correlation, 0 indicating no linear correlation and 1 indicating total positive linear correlation. Furthermore, *r* is invariant under separate changes in location and scale of the two variables, implying that for a linear function the angle to the x-axis does not affect *r*.

To calculate *r* for two variables *X* and *Y*, one divides the covariance of *X* and *Y* by the product of their standard deviations.
